# Six new species of terrestrial crabs (Crustacea, Decapoda, Potamidae) from Thailand

**DOI:** 10.3897/zookeys.1284.182481

**Published:** 2026-07-03

**Authors:** Zhi Wan Tan, Phaibul Naiyanetr, Darren C. J. Yeo

**Affiliations:** 1 Department of Biological Sciences, Faculty of Science, National University of Singapore, 16 Science Drive 4, Singapore 117558, Singapore Department of Biological Sciences, Faculty of Science, National University of Singapore Singapore Singapore; 2 Department of Biology, Faculty of Science, Chulalongkorn University, Bangkok 10330, Thailand Lee Kong Chian Natural History Museum, Faculty of Science, National University of Singapore Singapore Singapore; 3 Lee Kong Chian Natural History Museum, Faculty of Science, National University of Singapore, 2 Conservatory Drive, Singapore 117377, Singapore Department of Biology, Faculty of Science, Chulalongkorn University Bangkok Thailand

**Keywords:** Brachyura, Indochina, potamids, Potamiscinae, primary freshwater crab, taxonomy

## Abstract

A re-examination of old collections of terrestrially adapted primary freshwater crabs in the family Potamidae from various regions of Thailand, as well as aquarium trade material, revealed two new species from the genus *Thaiphusa* Ng & Naiyanetr, 1993, *T. reginamimus***sp. nov**. and *T. mongkol***sp. nov**. and four new species from the genus *Thaipotamon* Ng & Naiyanetr, 1993: *T. nandidarbhai***sp. nov**., *T. songkhwae***sp. nov**., *T. suvankorni***sp. nov**. and *T. wangsaphung***sp. nov**. Species of *Thaiphusa* and *Thaipotamon* are characterised by their inflated, ovoid carapace, but these can be distinguished from one another and other similarly rotund Thai potamids by suites of differences in gonopodal, third maxilliped and sternopleonal morphology. The new species are described and compared with congeners; they can be distinguished primarily by differences in the male gonopod morphology. Identification keys to all species of *Thaiphusa* and *Thaipotamon* are provided.

## Introduction

Thailand, which is part of the Indo-Burma biogeographic region, is a hotspot for primary freshwater crab diversity, where the family Potamidae is particularly well studied and represented (Yeo and Ng 1999; Cumberlidge et al. 2012; Yeo et al. 2014). To date, 78 species of potamid crab have been recorded from Thailand (Ng et al. 2008; Leelawathanagoon et al. 2010; Naiyanetr and Yeo 2010; Yeo and Naiyanetr 2010; Ng and Vidthayanon 2013; Promdam et al. 2014, 2017; Lheknim and Ng 2016; Takeda et al. 2019; Rogers et al. 2021; Tan et al. 2022, 2023, 2024; Lheknim et al. 2023; Tan and Yeo 2024). Potamid crabs in Thailand have adapted to a variety of habitats, with many species exhibiting a typically aquatic habit; often characterised by a flat and low carapace height and relatively stout ambulatory legs (e.g., *Demanietta* Bott, 1966, *Indochinamon* Yeo & Ng, 2007, and *Tomaculamon* Yeo & Ng, 1997). Conversely, several genera have adopted a more terrestrial or semiterrestrial lifestyle and are noted by their often highly inflated, globose carapace. Examples of such genera in Thailand include *Thaiphusa* Ng & Naiyanetr, 1993, *Thaipotamon* Ng & Naiyanetr, 1993, and *Pudaengon* Ng & Naiyanetr, 1995.

The genus *Thaiphusa* Ng & Naiyanetr, 1993, was established for three species, distributed from southern Myanmar and western Thailand to eastern Thailand, viz., *T. sirikit* (Naiyanetr, 1992) (type species, Kanchanaburi province, western Thailand), *T. chantaburiensis* (Chuensri, 1973) (Chanthaburi province, eastern Thailand), and *T. tenasserimensis* (De Man, 1898) (southern Myanmar) (see Ng and Naiyanetr 1993). The similarly globose-carapace genus, *Thaipotamon*, was also established by Ng and Naiyanetr (1993) and is currently represented by seven species, all endemic to Thailand, viz., *T. lomkao* Ng & Naiyanetr, 1993 (type species, Phetchabun province, north-central Thailand), *T. siamense* (A. Milne-Edwards, 1869) (Bangkok, central Thailand), *T. smitinandi* (Naiyanetr & Türkay, 1984) (Chanthaburi province, eastern Thailand), *T. dansai* Ng & Naiyanetr, 1993 (Loei province, northeastern Thailand), *T. varoonphornae* Ng & Naiyanetr, 1993 (Sa Kaeo province, eastern Thailand), *T. chulabhorn* Naiyanetr, 1993 (Maha Sarakham province, northeastern Thailand), and *T. holthuisi* Naiyanetr & Yeo, 2010 (Phetchabun province, north-central Thailand). These two globose-carapace genera can still be distinguished easily from one another by differences in the cervical and H-shaped grooves, external orbital tooth, position of the male sternopleonal cavity, male pleon, and their male first pleopod (gonopod 1 or G1) terminal article morphology (see Ng and Naiyanetr 1993 for detailed account).

**Figure 1.**
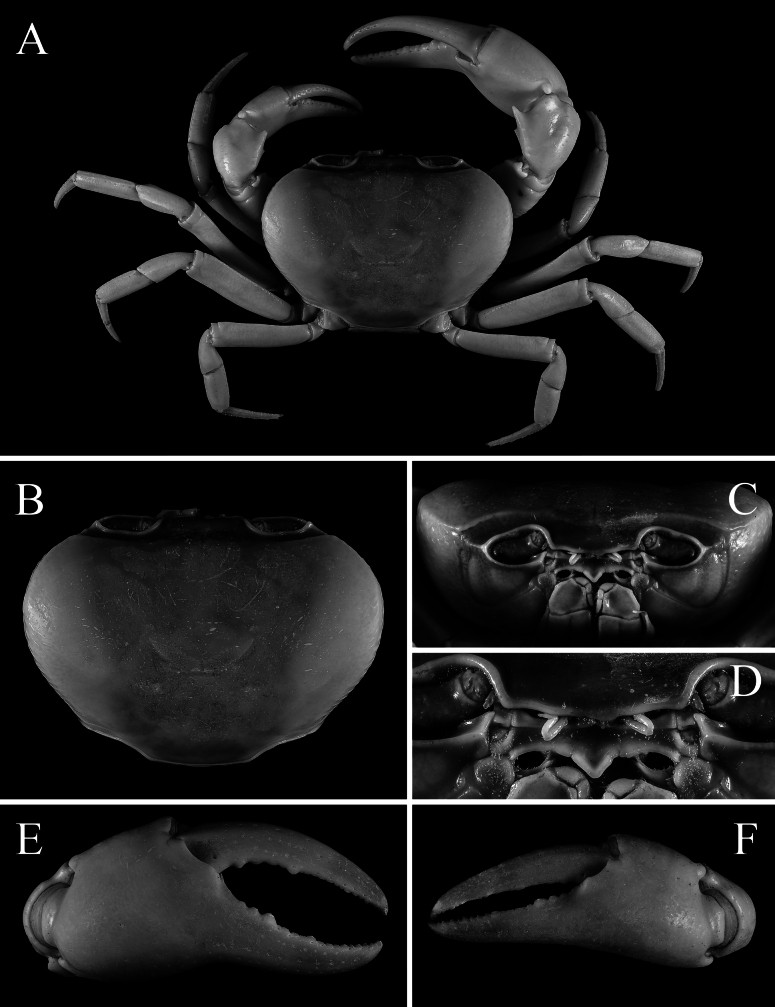
*Thaiphusa reginamimus* sp. nov., male, holotype (42.0 × 29.5 mm) (ZRC 2002.0109). **A**. Overall dorsal view; **B**. Cephalothorax (dorsal view); **C**. Cephalothorax (frontal view); **D**. Antennular fossa and epistome; **E**. Major chela; **F**. Minor chela.

**Figure 2.**
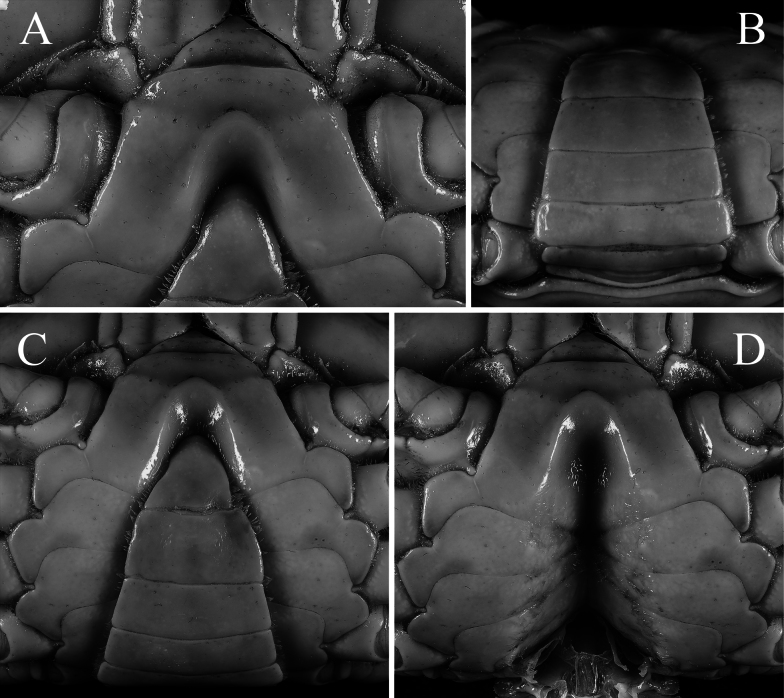
*Thaiphusa reginamimus* sp. nov., male, holotype (42.0 × 29.5 mm) (ZRC 2002.0109). **A**. Anterior thoracic sternites; **B**. Pleon somites 1–5; **C**. Posterior thoracic sternites, pleonal somites 4–6, and telson; **D**. Sternopleonal cavity.

**Figure 3.**
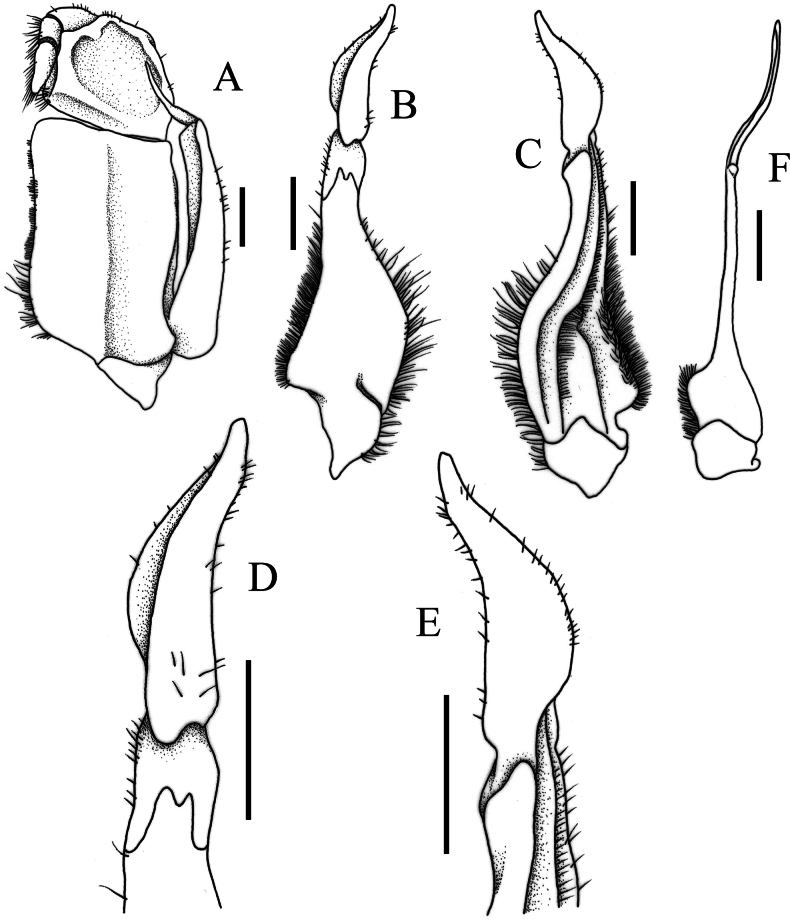
*Thaiphusa reginamimus* sp. nov., male, holotype (42.0 × 29.5 mm) (ZRC 2002.0109). **A**. Left third maxilliped; **B**. Right G1 (dorsal view); **C**. Right G1 (ventral view); **D**. Terminal article of right G1 (dorsal view); **E**. Terminal article of right G1 (ventral view); **F**. Right G2. Scale bars: 2 mm.

**Figure 4.**
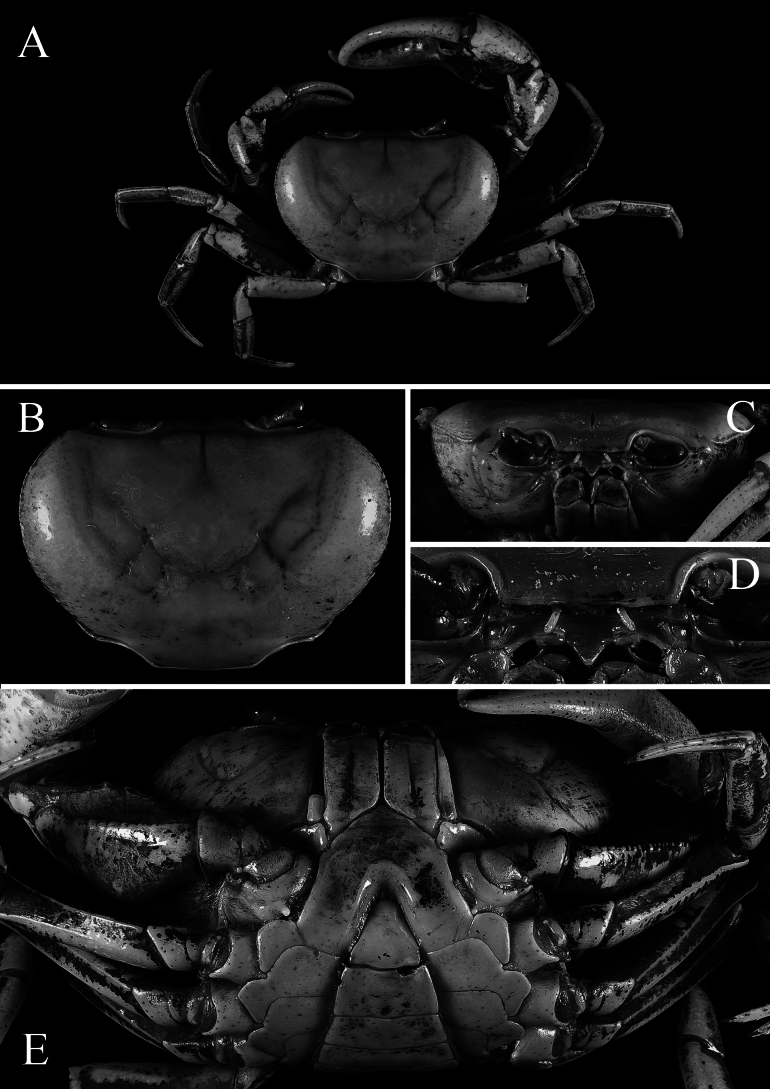
*Thaiphusa sirikit* (Naiyanetr, 1992), male, paratype (46.7 × 32.2 mm) (ZRC 1991.1880). **A**. Overall dorsal view; **B**. Cephalothorax (dorsal view); **C**. Cephalothorax (frontal view); **D**. Antennular fossa and epistome; **E**. Ventral view.

**Figure 5.**
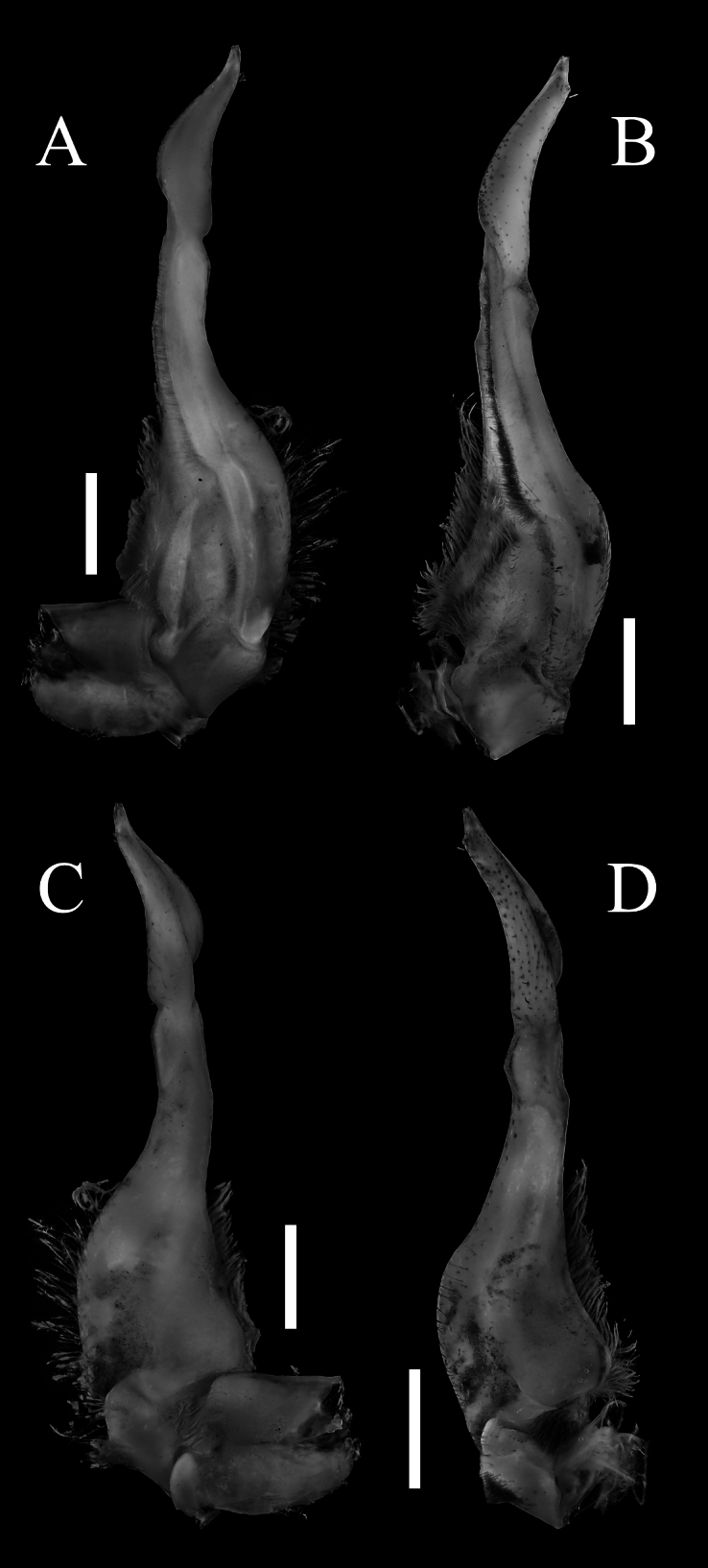
G1s. **A, C**. *Thaiphusa reginamimus* sp. nov., male, holotype (42.0 × 29.5 mm) (ZRC 2002.0109); **B, D**. *Thaiphusa sirikit* (Naiyanetr, 1992), male, paratype (46.7 × 32.2 mm) (ZRC 1991.1880). **A, B**. Ventral view; **C, D**. Dorsal view. Scale bars: 2 mm.

**Figure 6.**
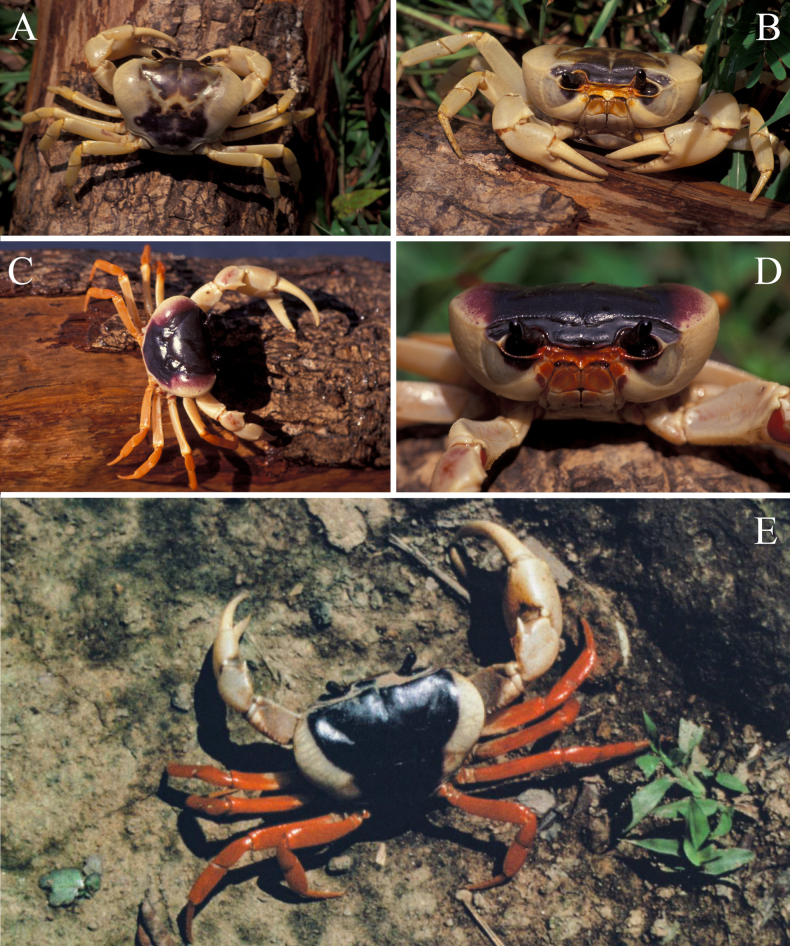
Live colouration. **A–D**. *Thaiphusa reginamimus* sp. nov.; **E**. *Thaiphusa sirikit* (Naiyanetr, 1992) (reproduced from Naiyanetr 1992).

In the mid-1990s, specimens of a *Thaiphusa* species closely resembling *T. sirikit* (known in Thailand as the Regal crab) appeared in the aquarium retail trade in Singapore. Subsequent closer examination of material purchased from these shops revealed that they in fact belonged to a distinct and new species, although specific locality data for the specimens was lacking. According to aquarium wholesalers in Singapore at the time, the material was imported from Thailand. Various Thai field collector sources further confirmed that similar-looking crabs were occasionally collected for the aquarium trade from the wild in Kanchanaburi province, western Thailand, narrowing the search area for the likely locality, though the precise location remained unknown. Kamphol Udomritthiruj managed to trace the original collector and exporter of these crabs, and the provenance of the new species was finally established sometime in mid-2017. The collector confirmed that the collection site was in Kanchanaburi province and provided details. This provided the present study with the necessary information to now describe formally the new species, herein named *Thaiphusa reginamimus* sp. nov. It is distinguished from *T. sirikit*, which it most closely resembles, by various carapace and male first pleopod characters, as well as by its slightly different live colouration and colour pattern (Figs 1, 2, 3, 4, 5, 6).

**Figure 7.**
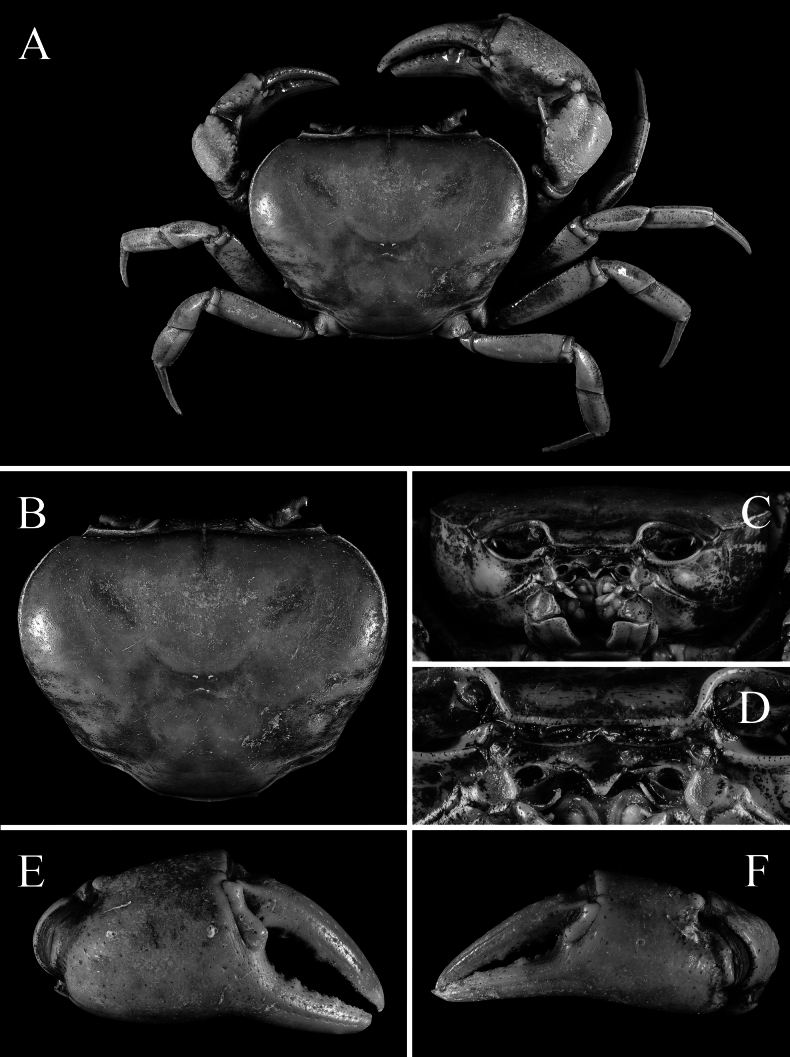
*Thaiphusa mongkol* sp. nov., male, holotype (38.3 × 29.3 mm) (ZRC 2024.0331). **A**. Overall dorsal view; **B**. Cephalothorax (dorsal view); **C**. Cephalothorax (frontal view); **D**. Antennular fossa and epistome; **E**. Major chela; **F**. Minor chela.

**Figure 8.**
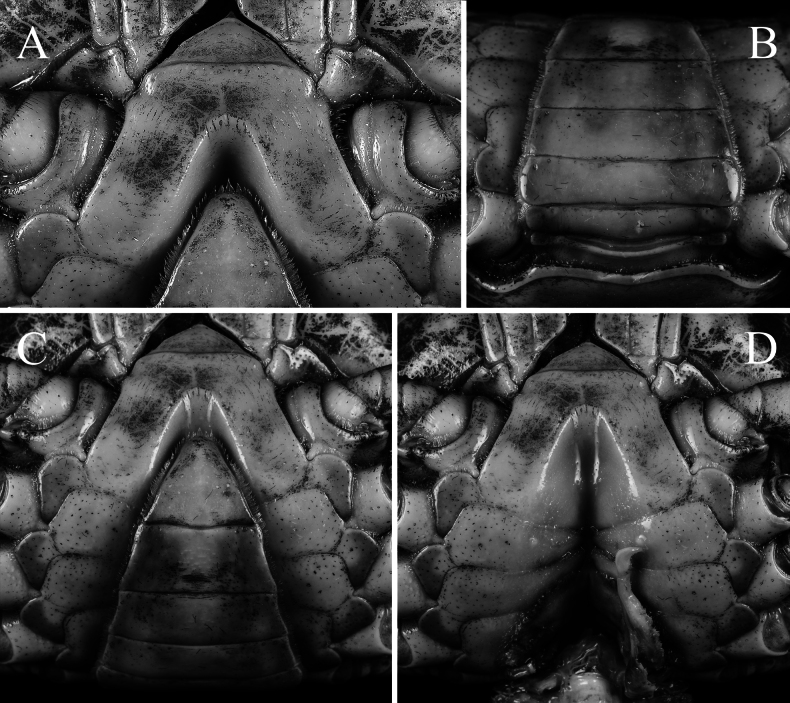
*Thaiphusa mongkol* sp. nov., male, holotype (38.3 × 29.3 mm) (ZRC 2024.0331). **A**. Anterior thoracic sternites; **B**. Pleon somites 1–5; **C**. Posterior thoracic sternites, pleonal somites 4–6; **D**. Sternopleonal cavity, with left gonopods in situ.

**Figure 9.**
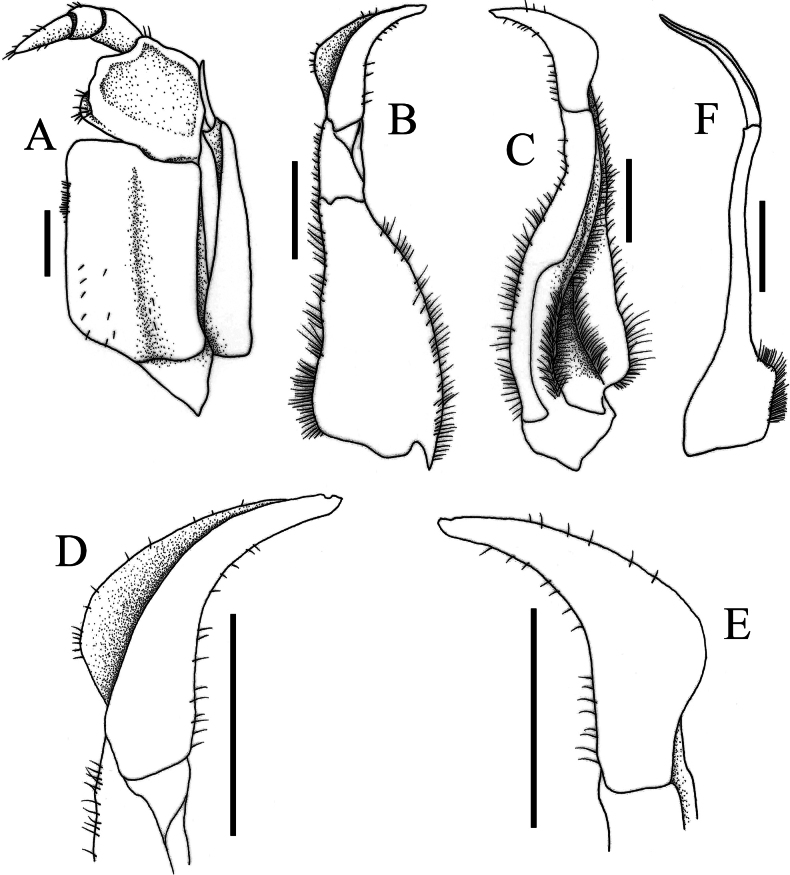
*Thaiphusa mongkol* sp. nov., male, holotype (38.3 × 29.3 mm) (ZRC 2024.0331). **A**. Left third maxilliped; **B**. Right G1 (dorsal view); **C**. Right G1 (ventral view); **D**. Terminal article of right G1 (dorsal view); **E**. Terminal article of right G1 (ventral view); **F**. Right G2. Scale bars: 2 mm.

**Figure 10.**
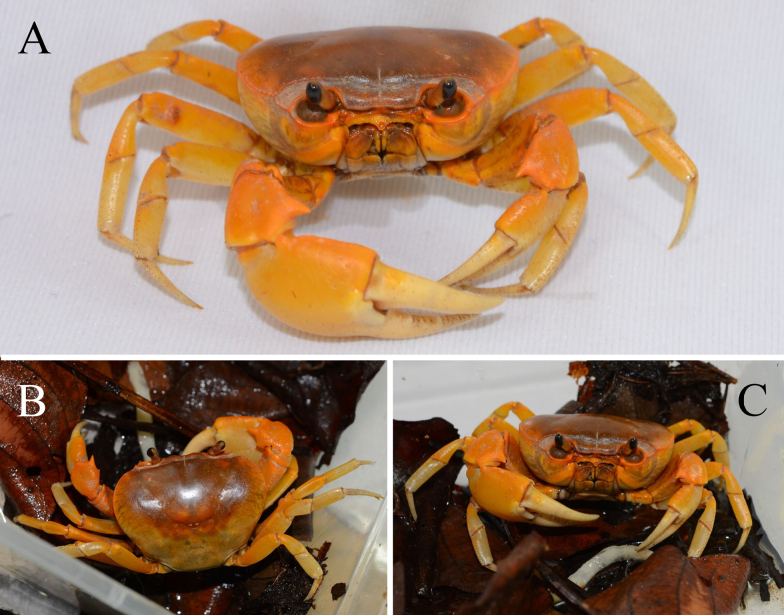
Live colouration. **A–C**. *Thaiphusa mongkol* sp. nov. (photo credit Kamonchanok Wongissarakul, THNHM).

**Figure 11.**
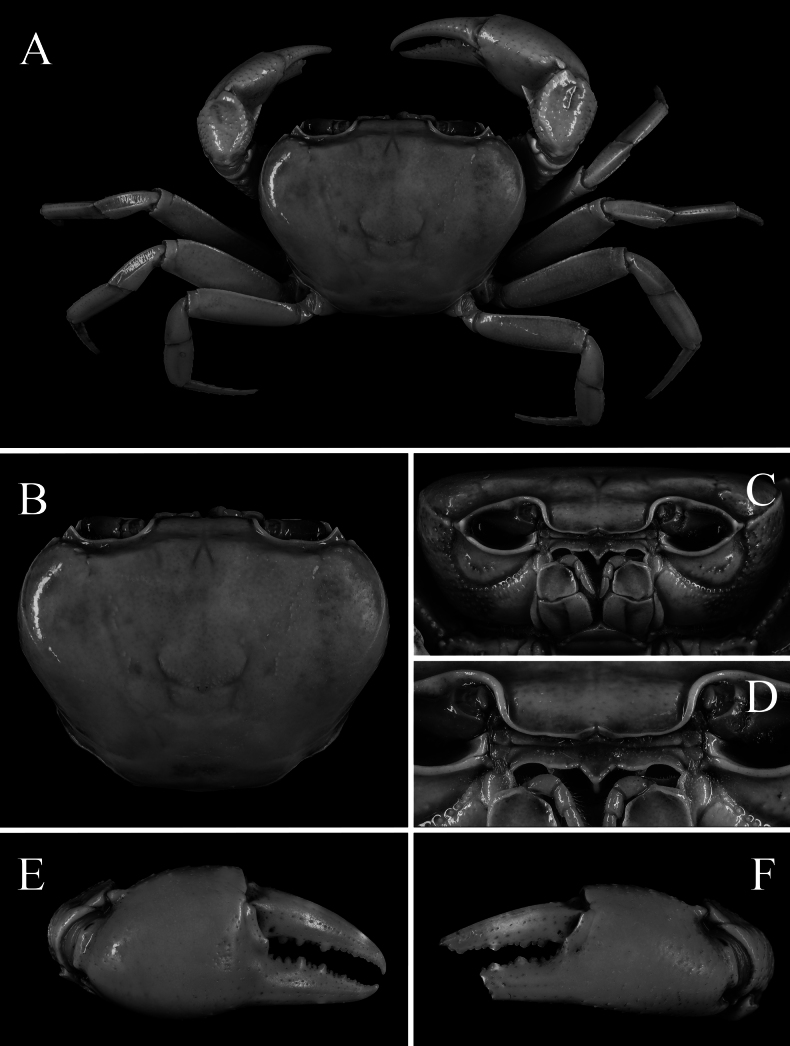
*Thaipotamon nandidarbhai* sp. nov., male, holotype (38.7 × 29.6 mm) (ZRC 2024.0333). **A**. Overall dorsal view; **B**. Cephalothorax (dorsal view); **C**. Cephalothorax (frontal view); **D**. Antennular fossa and epistome; **E**. Major chela; **F**. Minor chela.

**Figure 12.**
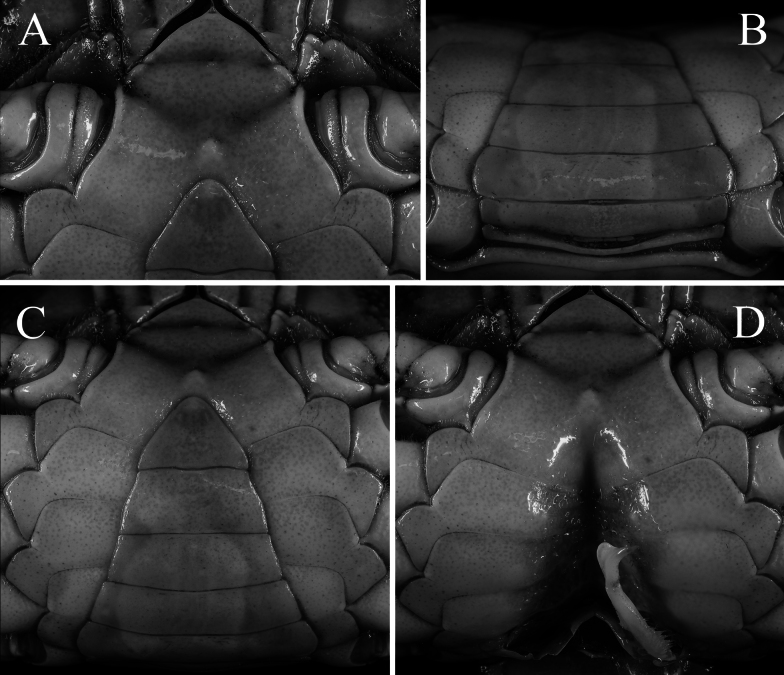
*Thaipotamon nandidarbhai* sp. nov., male, holotype (38.7 × 29.6 mm) (ZRC 2024.0333). **A**. Anterior thoracic sternites; **B**. Pleon somites 1–5; **C**. Posterior thoracic sternites, pleonal somites 3–6, and telson; **D**. Sternopleonal cavity, showing left G1.

**Figure 13.**
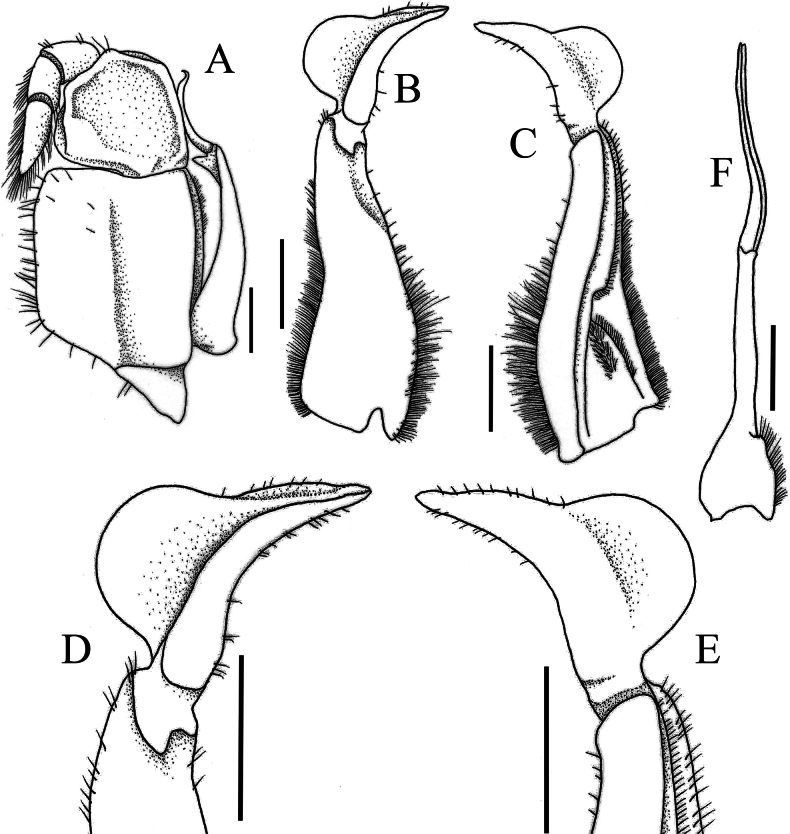
*Thaipotamon nandidarbhai* sp. nov., male, holotype (38.7 × 29.6 mm) (ZRC 2024.0333). **A**. Left third maxilliped; **B**. Right G1 (dorsal view); **C**. Right G1 (ventral view); **D**. Terminal article of right G1 (dorsal view); **E**. Terminal article of right G1 (ventral view); **F**. Right G2. Scale bars: 2 mm.

**Figure 14.**
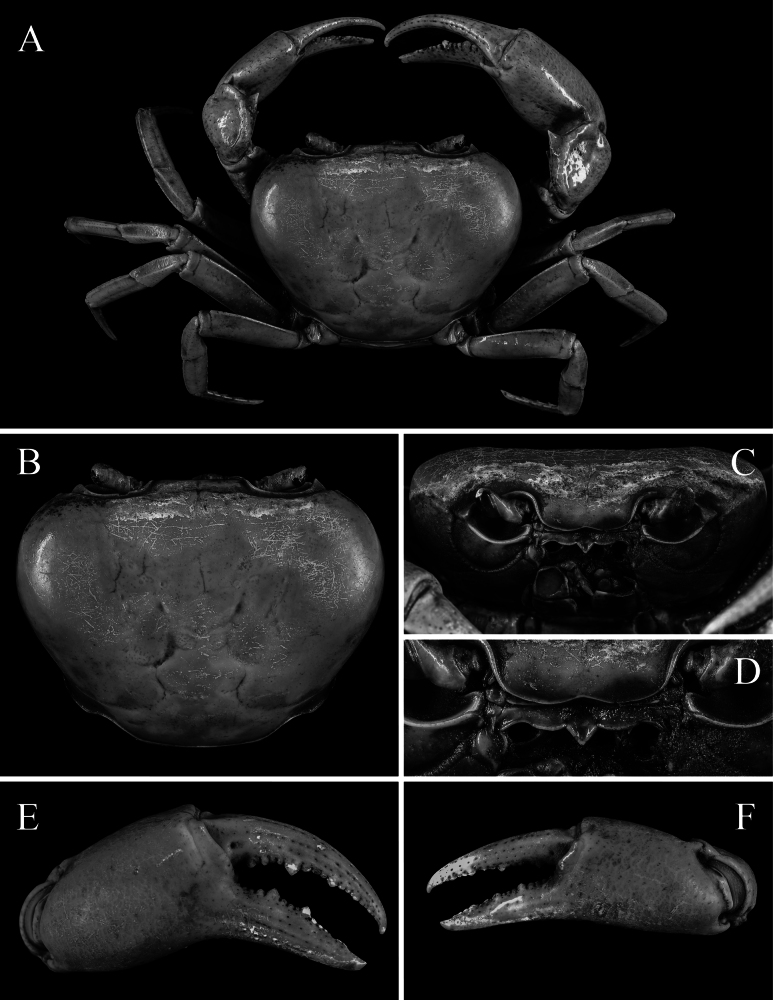
*Thaipotamon songkhwae* sp. nov., male, holotype (58.0 × 42.0 mm) (ZRC 2024.0334). **A**. Overall dorsal view; **B**. Cephalothorax (dorsal view); **C**. Cephalothorax (frontal view); **D**. Antennular fossa and epistome; **E**. Major chela; **F**. Minor chela.

**Figure 15.**
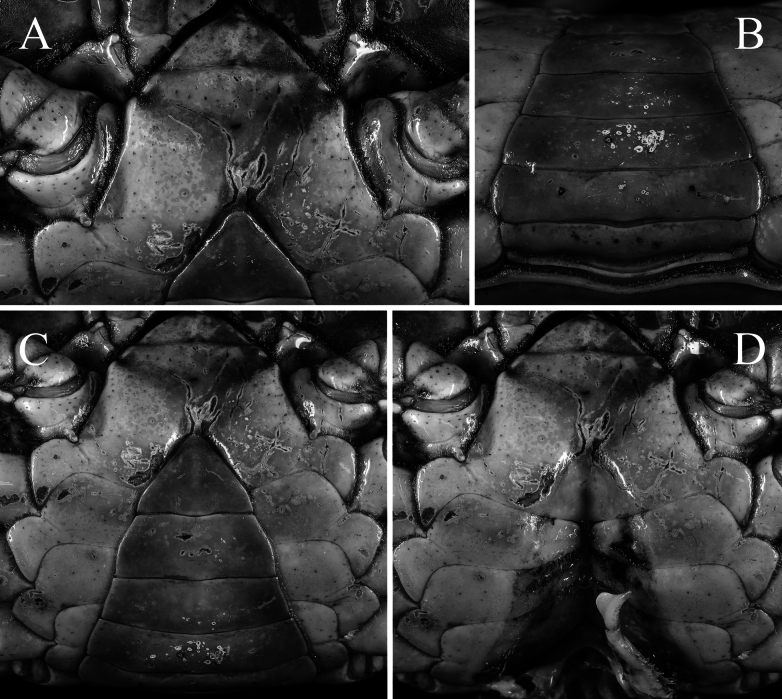
*Thaipotamon songkhwae* sp. nov., male, holotype (58.0 × 42.0 mm) (ZRC 2024.0334). **A**. Anterior thoracic sternites; **B**. Pleon somites 1–5; **C**. Posterior thoracic sternites, pleonal somites 3–5, and telson; **D**. Sternopleonal cavity, showing left G1.

**Figure 16.**
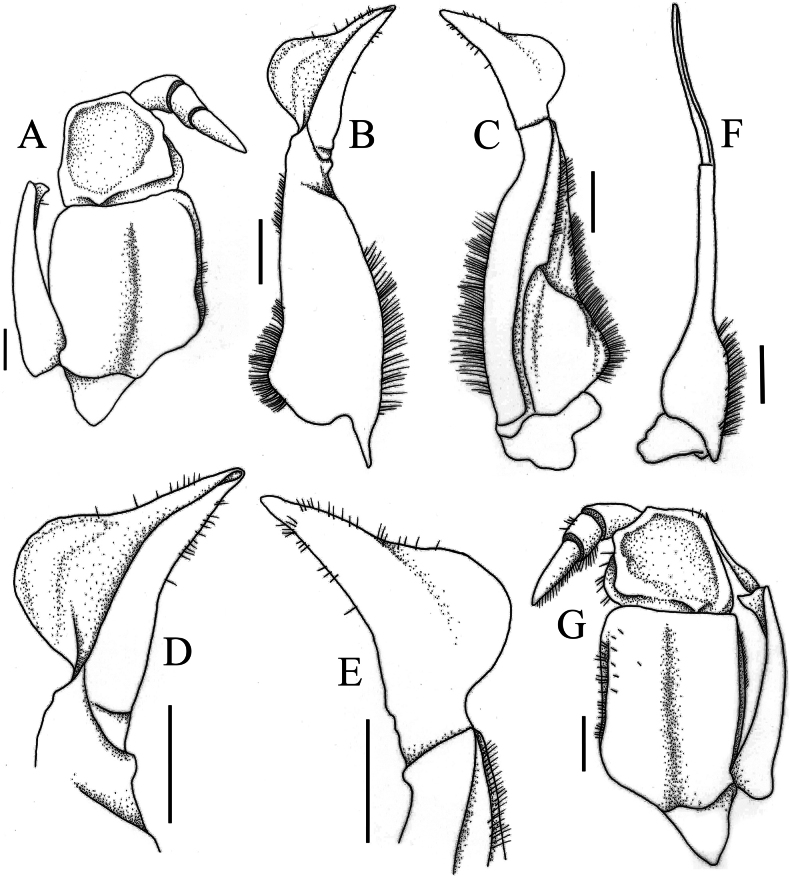
*Thaipotamon songkhwae* new species, **A–F**. Male, holotype (58.0 × 42.0 mm) (ZRC 2024.0334); **G**. Male, paratype (49.6 × 36.5 mm) (ZRC 2024.0335). **A**. Right third maxilliped; **B**. Right G1 (dorsal view); **C**. Right G1 (ventral view); **D**. Terminal article of right G1 (dorsal view); **E**. Terminal article of right G1 (ventral view); **F**. Right G2; **G**. Left third maxilliped. Scale bars: 2 mm.

**Figure 17.**
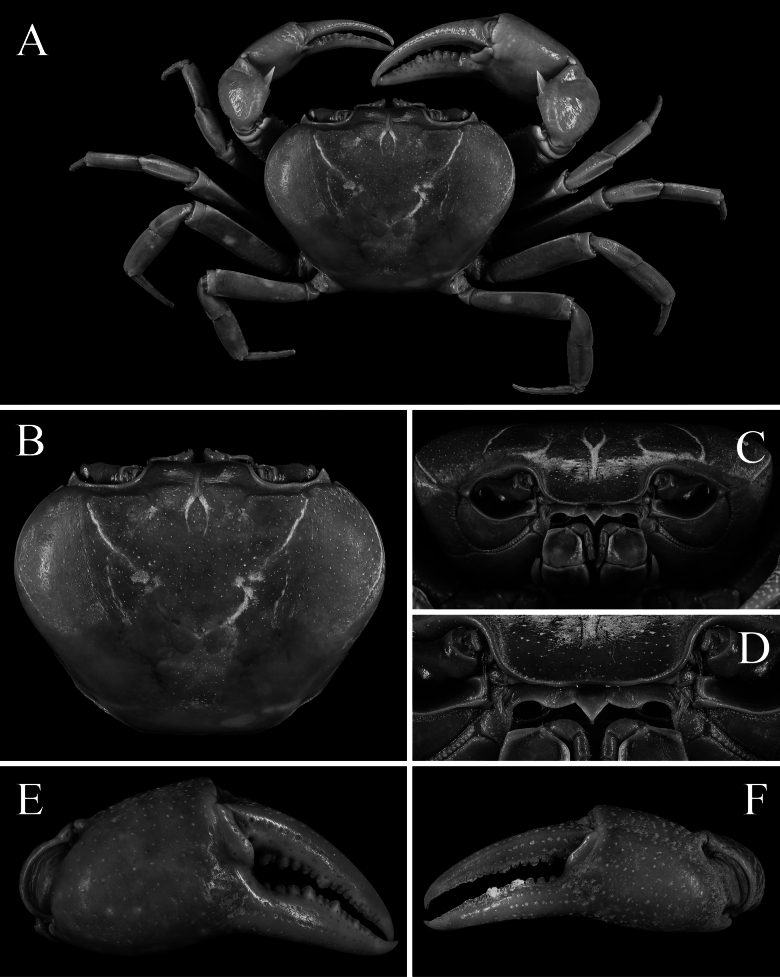
*Thaipotamon suvankorni* sp. nov., male, holotype (45.5 × 35.0 mm) (ZRC 2024.0336). **A**. Overall dorsal view; **B**. Cephalothorax (dorsal view); **C**. Cephalothorax (frontal view); **D**. Antennular fossa and epistome; **E**. Major chela; **F**. Minor chela.

**Figure 18.**
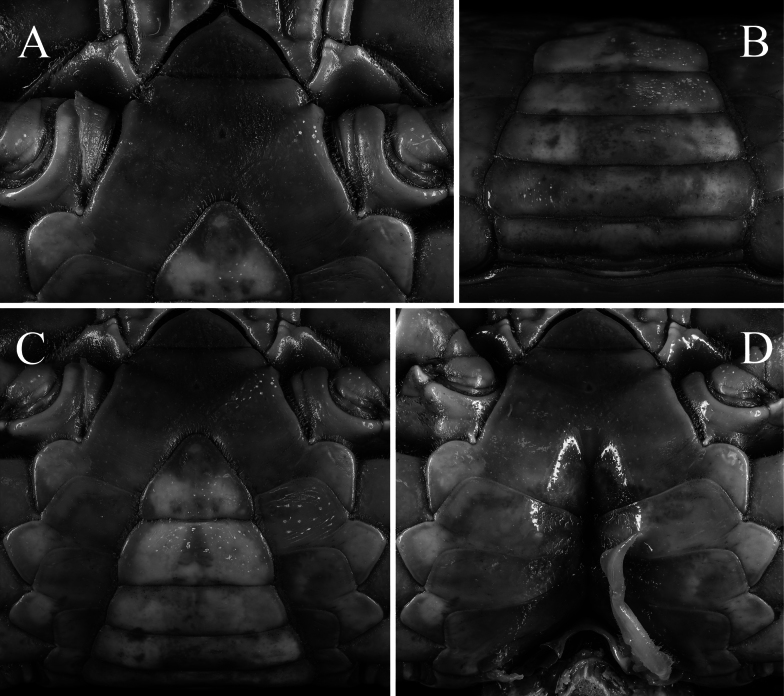
*Thaipotamon suvankorni* sp. nov., male, holotype (45.5 × 35.0 mm) (ZRC 2024.0336). **A**. Anterior thoracic sternites; **B**. Pleon somites 1–5; **C**. Posterior thoracic sternites, pleonal somites 4–6, and telson; **D**. Sternopleonal cavity, showing left G1.

**Figure 19.**
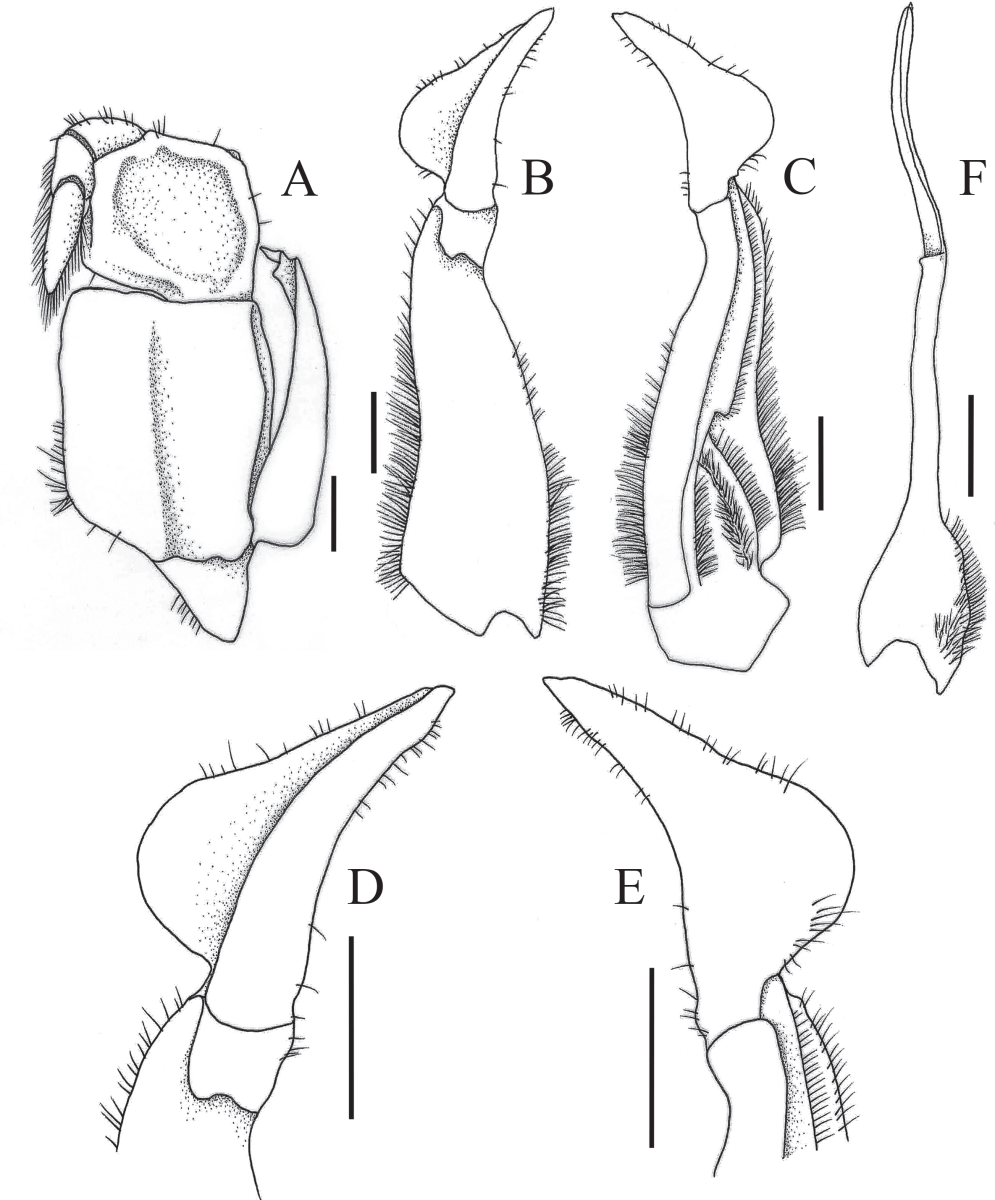
*Thaipotamon suvankorni* sp. nov., male, holotype (45.5 × 35.0 mm) (ZRC 2024.0336). **A**. Left third maxilliped; **B**. Right G1 (dorsal view); **C**. Right G1 (ventral view); **D**. Terminal article of right G1 (dorsal view); **E**. Terminal article of right G1 (ventral view); **F**. Right G2. Scale bars: 2 mm.

**Figure 20.**
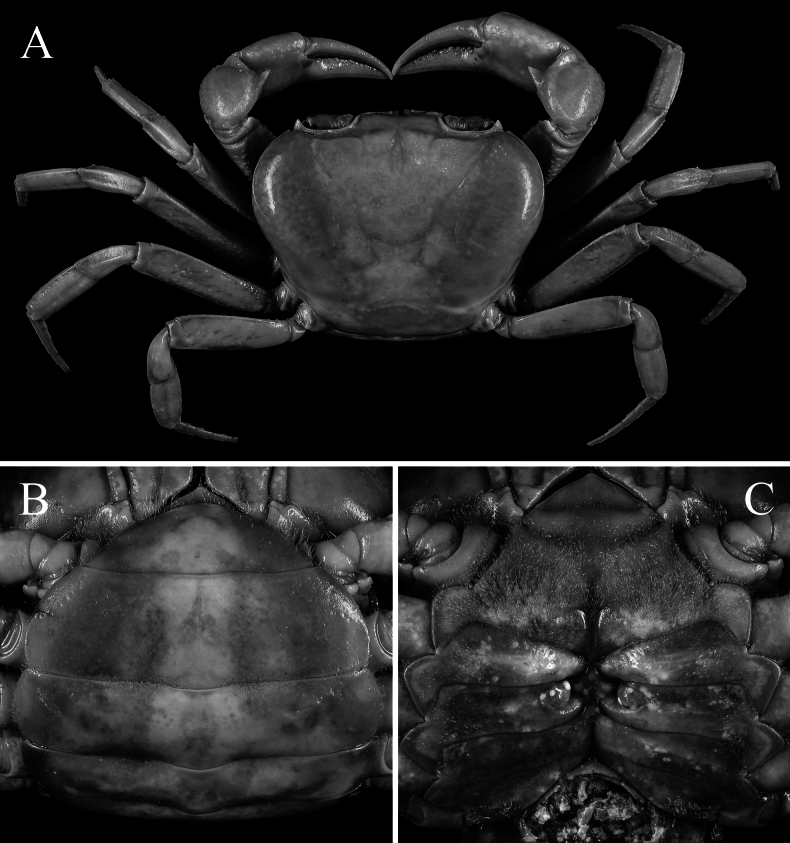
*Thaipotamon suvankorni* sp. nov., female, paratype (41.3 × 32.3 mm) (ZRC 2024.0337). **A**. Overall dorsal view; **B**. Pleonal somites 4–6 and telson; **C**. Sternopleonal cavity, showing vulvae.

**Figure 21.**
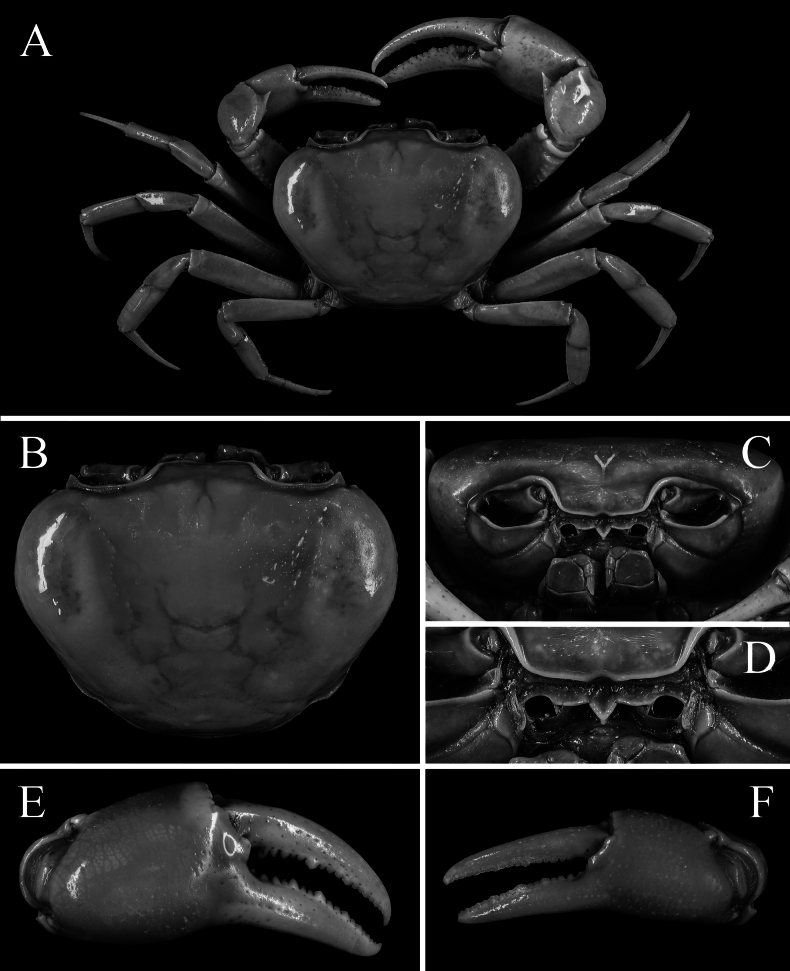
*Thaipotamon wangsaphung* sp. nov., male, holotype (40.7 × 28.7 mm) (ZRC 2024.0338). **A**. Overall dorsal view; **B**. Cephalothorax (dorsal view); **C**. Cephalothorax (frontal view); **D**. Antennular fossa and epistome; **E**. Major chela; **F**. Minor chela.

**Figure 22.**
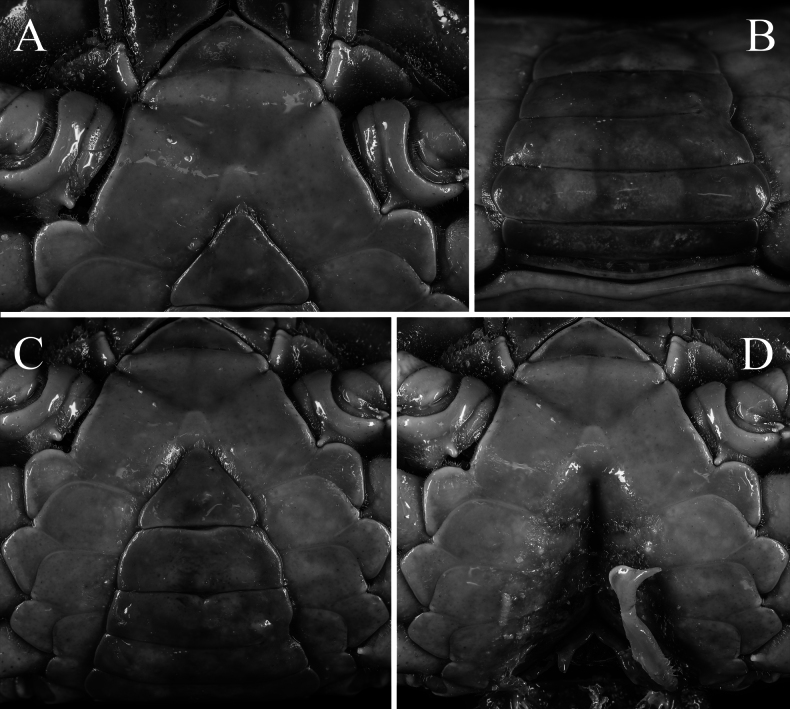
*Thaipotamon wangsaphung* sp. nov., male, holotype (40.7 × 28.7 mm) (ZRC 2024.0338). **A**. Anterior thoracic sternites; **B**. Pleon somites 1–5; **C**. Posterior thoracic sternites, pleonal somites 4–6; **D**. Sternopleonal cavity, showing left G1.

**Figure 23.**
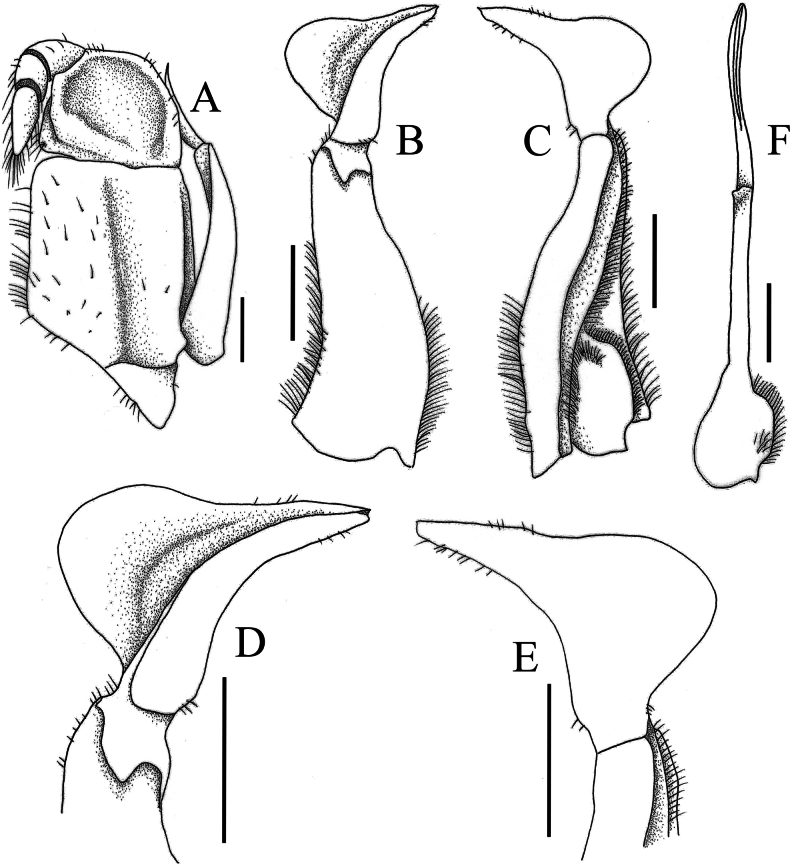
*Thaipotamon wangsaphung* sp. nov., male, holotype (40.7 × 28.7 mm) (ZRC 2024.0338). **A**. Left third maxilliped; **B**. Right G1 (dorsal view); **C**. Right G1 (ventral view); **D**. Terminal article of right G1 (dorsal view); **E**. Terminal article of right G1 (ventral view); **F**. Right G2. Scale bars: 2 mm.

**Figure 24.**
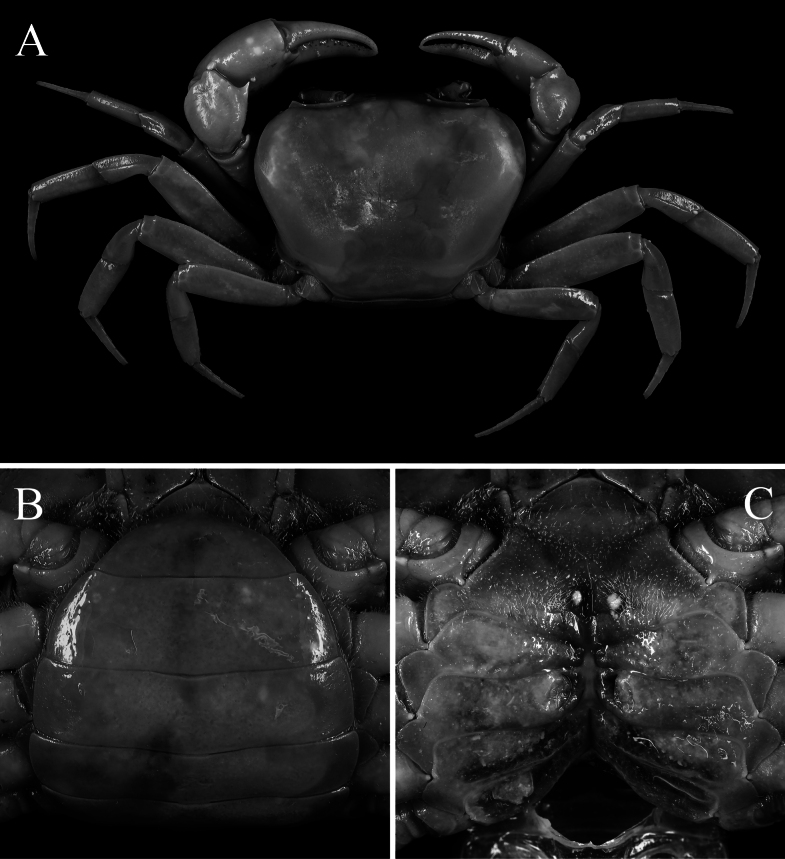
*Thaipotamon wangsaphung* sp. nov., female, paratype (33.0 × 24.9 mm) (ZRC 2024.0339). **A**. Overall dorsal view; **B**. Pleonal somites 4–6 and telson; **C**. Sternopleonal cavity, showing vulvae.

At the same time, comparisons and re-examination were conducted on other potamid terrestrial crab specimens collected from various localities across Thailand in the 1990s. In addition to *Thaiphusa reginamimus* sp. nov., the present study recognised another new species of *Thaiphusa* (Figs 7, 8, 9, 10) and four new species of *Thaipotamon* (Figs 11, 12, 13, 14, 15, 16, 17, 18, 19, 20, 21, 22, 23, 24), which are described in the present paper, bringing the total number of species of *Thaiphusa* and *Thaipotamon* to five and 11, respectively. The species of *Thaiphusa* and *Thaipotamon* generally resemble their respective congeners in external morphology, with the G1 structure being the main and most reliable distinguishing feature. As such, their taxonomy covered here is heavily reliant on the G1 structure, with external carapace features used as secondary supporting characters. Identification keys to the species of *Thaiphusa* and *Thaipotamon*, based on adult males, are also provided.

## Materials and methods

The crabs are preserved in 75% ethanol and deposited in the Zoological Reference Collection of the Lee Kong Chian Natural History Museum, National University of Singapore (**ZRC**) and the collections at the Thailand Natural History Museum (**THNHM**), National Science Museum, Thailand. The morphological terminology used in the descriptive accounts follows Ng (1988), with updates following Guinot et al. (2013) and Davie et al. (2015). Measurements are of the carapace maximum width (**CW**) and length at the midline (**CL**). All measurements are in millimetres (mm), taken using dial callipers. Specimens were examined with the Leica M80 and M205c stereomicroscope. Camera lucida illustrations were made using the drawing tube mounted on these stereomicroscopes. Photographs of specimens were taken using the Olympus OM-D E-M1 Mark III mirrorless interchangeable lens camera with M.Zuiko Digital ED 60 mm F2.8 lens. Figures were edited and assembled using Adobe Photoshop Lightroom and Adobe Photoshop. The following abbreviations are used: **G1** = gonopod 1 (male first pleopod); **G2** = gonopod 2 (male second pleopod).

## Taxonomy

**Family Potamidae Ortmann, 1896**

**Subfamily Potamiscinae Bott, 1970**

***Thaiphusa* Ng & Naiyanetr, 1993**

### Thaiphusa reginamimus sp. nov.

Taxon classificationAnimaliaDecapodaPotamidae

B31A9CD6-AC4D-5F6C-B4FF-1D7B94084C12

https://zoobank.org/198F0200-BA1A-460B-85F8-AC198CDE42A4

Figs 1, 2, 3, 5A, C, 6

#### Type material.

***Holotype***. Thailand • male (42.0 × 29.5 mm); aquarium material purchased in Singapore by S.H. Tan on 24 Sep. 1996, originally collected in Kanchanaburi Province, Maenam Noi, near Ban Huai; Kittipong Jarutanin leg.; 1996; ZRC 2002.0109. ***Paratypes***. Thailand • 3 males (41.3 × 28.9, 41.0 × 29.2, 41.0 × 28.8 mm); 5 females (46.4 × 32.3–40.9 × 28.3 mm); same collection data as for holotype; ZRC 2002.0110.

#### Non-type specimens.

Thailand • 1 male (38.0 × 26.6 mm); 1 female (45.8 × 31.3 mm); aquarium material purchased in Singapore by S.H. Tan on Oct. 1996; ZRC 1997.656 • 1 male (37.4 × 26.4 mm); Kanchanaburi Province, Thong Pha Phum District; Sunchai Makchai leg.; 2013; THNHM-IV-13089 • 1 female (39.0 × 28.0 mm); same collection data as for previous; THNHM-IV-13090 • 1 female (38.7 × 27.9 mm); same collection data as for previous; THNHM-IV-13091 • 1 female (34.1 × 24.2 mm); same collection data as for previous; THNHM-IV-13092.

#### Diagnosis.

Carapace (Fig. 1A, B) transversely ovate, distinctly wider than long, high; dorsal surface (Fig. 1C) strongly convex longitudinally, distinctly inflated transversely, glabrous, smooth. Epigastric, postorbital cristae (Fig. 1A, B) smooth, confluent; external orbital tooth outer margin distinctly longer than inner margin; epibranchial tooth low, blunt, confluent with postorbital cristae; anterolateral margin rounded, indistinct; epistome posterior margin (Fig. 1D) median tooth relatively broadly triangular, outer parts gently concave. Third maxilliped (Fig. 3A) exopod flagellum subequal to half of merus width. Ambulatory dactyli (Fig. 1A) elongated, slender. Male thoracic sternite 3/4 groove demarcating suture prominent (Fig. 2B–D). Male pleon (Fig. 2B, C) narrowly triangular; pleonal somite 6 transversely rectangular, much wider than long, lateral margins convex. G1 terminal article (Fig. 3B–E) sinuous, relatively long, 0.52 × length of subterminal article, tip truncate, with low, broad dorsal flap, with broad, gently convex apex; subterminal article gradually narrowing from broad proximal part into slender, neck-like distal part, without forming shelf on outer margin, with distal part 0.49 × length of proximal part.

#### Etymology.

The species name is derived from the combination of *Regina* and *mimus*, queen and mimic in Latin, respectively, due to the similarity in morphology and colouration to *Thaiphusa sirikit*, which was named in honour of the late Queen and Queen Mother of Thailand. Name used as a noun in apposition.

#### Remarks.

*Thaiphusa reginamimus* sp. nov., is closest in external morphology to *T. sirikit*, which also occurs in Sai Yok and Thong Pha Phum districts, Kanchanaburi Province. In addition, its striking purple and white dorsal carapace colours also closely resemble those of *T. sirikit*. *Thaiphusa reginamimus* sp. nov., however, is distinguishable from *T. sirikit* by the following morphological characters: male thoracic sternite 3/4 groove demarcating suture prominent (Fig. 2A, C) (vs male thoracic sternite 3/4 groove demarcating suture not distinct, Fig. 4E; cf. Ng and Naiyanetr 1993: fig. 22C); G1 terminal article proportionally shorter, ~ 0.5 × length of subterminal article (Figs 3B, 3C, 5A, 5C) (vs G1 terminal article proportionally longer, ~ 0.6 × length of subterminal article, Fig. 5B, D; cf. Ng and Naiyanetr 1993: fig. 56B, C); G1 terminal article dorsal flap relatively higher and more strongly convex, appearing relatively shorter longitudinally (Figs 3D, 3E, 5A, 5C) (vs G1 terminal article dorsal flap lower and more gently convex, appearing relatively longer longitudinally, Fig. 5B, D; cf. Ng and Naiyanetr 1993: fig. 56D, E); and G1 subterminal article with narrowed, neck-like distal part subequal to or shorter than expanded proximal part (Figs 3B, 3C, 5A, 5C) (vs G1 subterminal article with narrowed, neck-like distal part longer than expanded proximal part, Fig. 5B, D; cf. Ng and Naiyanetr 1993: fig. 56B–E). In addition, the live colouration of *T. reginamimus* sp. nov., varies and differs slightly from *T. sirikit* in that the carapace can exhibit a comparatively smaller, less defined dark patch, with the ambulatory legs that are paler in colouration, ranging from light orange to pale yellow (Fig. 6A–D) (vs a carapace that has very strongly defined black and ivory coloured regions, with brightly coloured, ambulatory legs, usually red, sometimes orange in colour; Fig. 6E; cf. Naiyanetr 1992: pl. 1).

#### Distribution.

Sai Yok and Thong Pha Phum districts, Kanchanaburi province, western Thailand.

#### Comparative material.

*Thaiphusa sirikit* (Naiyanetr, 1992) ***paratype***. Thailand • male (46.7 × 32.2 mm); Kanchanaburi Province, Sai Yok District, Ban Nam Chon; P. Naiyanetr leg.; Nov. 1987; ZRC 1991.1880.

### Thaiphusa mongkol sp. nov.

Taxon classificationAnimaliaDecapodaPotamidae

BF0E9F26-C630-5939-8E7E-4B1637104CD9

https://zoobank.org/2D5E5523-E212-4A35-A8E0-420A8222E50A

Figs 7, 8, 9, 10

#### Type material.

***Holotype***. Thailand • male (38.3 × 29.3 mm); Kanchanaburi Province, Thong Pha Phum District, Ban Pu Tae; Mongkol Wongkalasin leg.; 12 Aug. 1999; ZRC 2024.0331. ***Paratypes***. Thailand • 2 males (larger male 36.5 × 28.5 mm); same collection data as for holotype; ZRC 2024.0332 • 8 males (42.3 × 31.9, 41.7 × 32.0, 39.3 × 30.2, 37.4 × 28.9, 35.8 × 27.5, 35.5 × 28.0, 33.7 × 26.2, 33.7 × 26.2 mm); 3 females (40.8 × 31.9, 37.2 × 29.4, 34.2 × 26.6 mm); Kanchanaburi Province, Thong Pha Phum District, swamp forest, Weeyawat Jaitrong leg.; 30 May 2019; THNHM-IV-20172 • 1 male (36.8 × 28.5 mm); Kanchanaburi Province, Thong Pha Phum District; Sunchai Makchai leg.; 6 Nov. 2018; THNHM-IV-20173.

#### Diagnosis.

Carapace (Fig. 7A, B) transversely ovate, distinctly wider than long, high; dorsal surface (Fig. 7C) strongly convex, inflated transversely, longitudinally, glabrous, smooth. Epigastric, postorbital cristae (Fig. 7A, B) smooth, confluent; external orbital tooth outer margin concave, longer than inner margin; epibranchial tooth low, blunt, confluent with postorbital cristae; anterolateral margin rounded, subcristate; epistome posterior margin (Fig. 7D) median tooth relatively broadly triangular, outer parts gently concave. Third maxilliped (Fig. 9A) exopod flagellum half of merus width. Ambulatory dactyli (Fig. 7A) elongated, slender. Male pleon (Fig. 8B, C) narrowly triangular; pleonal somite 6 transversely rectangular, much wider than long, lateral margins sinuous. G1 terminal article (Fig. 9B–E) strongly bent outwards, relatively long, ~ 0.5 × length of subterminal article, tip tapered, with well-produced, broad dorsal flap for most part of terminal article, with broad gently convex apex at proximal third; subterminal article gradually narrowing from broad proximal part into slender, neck-like distal part, without cleft on outer margin, with distal part ~ 0.3 × length of proximal part.

#### Etymology.

The species is named after the collector of the holotype, Mr. Mongkol Wongkalasin. Used as noun in apposition.

#### Remarks.

*Thaiphusa mongkol* sp. nov., most closely resembles *T. chantaburiensis* in the G1 terminal article being distinctly bent outwards at the median part as opposed to the G1 terminal article being gently sinuous or almost straight in other *Thaiphusa* species. *Thaiphusa mongkol* sp. nov., however, can still be distinguished from *T. chantaburiensis* by the G1 terminal article that is more strongly bent outwards almost to an angle of 90° (Fig. 9B–E), as compared to the G1 terminal article that is less strongly bent outwards, to only ~ 70° (cf. Chuensri 1973: fig. 6; Ng and Naiyanetr 1993: figs 18, 19). In addition, the proximal part of the G1 terminal article of *T. mongkol* sp. nov., is without a lobed-shape bulge, and the dorsal flap is long, broad, with a gently convex apex in the proximal third (Fig. 9D, E). This contrasts with that of *T. chantaburiensis*, which possesses a lobed-shaped bulge at the proximal part and a dorsal flap with a bluntly angular apex in the proximal third (cf. Chuensri 1973: fig. 6; Ng and Naiyanetr 1993: figs 18, 19). Moreover, *Thaiphusa mongkol* sp. nov., is currently known only from Thong Pha Phum district in Kanchanaburi Province, western Thailand, which is more than 400 km away from the locality of *T. chantaburiensis*, in Chanthaburi Province, eastern Thailand.

In life, *Thaiphusa mongkol* sp. nov., ranges in colouration, with a carapace that sports a uniformly bright yellow-orange colour (Fig. 10). This colouration is immediately different from both *T. sirikit* and *T. reginamimus* sp. nov., in the same area, which has a more distinctly patterned carapace, mainly ivory/cream in colour with a dark central patch (Fig. 6).

#### Distribution.

Thong Pha Phum district, Kanchanaburi province, western Thailand.

##### *Thaipotamon* Ng & Naiyanetr, 1993

### Thaipotamon nandidarbhai sp. nov.

Taxon classificationAnimaliaDecapodaPotamidae

49134F33-CB29-554A-93E9-D712007E6298

https://zoobank.org/CEFFD61A-099B-4AC5-8E2A-02DF5D644421

Figs 11, 12, 13

Thaipotamon nandidarbhi Naiyanetr, in Nandidarbha and Nandidarbha 2001 [nomen nudum].

#### Type material.

***Holotype***. Thailand • male (38.7 × 29.6 mm); Phrae Province, Sung Men District; Sermsak Nandidarbha leg.; Oct. 1997; ZRC 2024.0333.

#### Diagnosis.

Carapace (Fig. 11A, B) transversely sub-ovate, wider than long, CW/CL ratio 1.30, high; dorsal surface (Fig. 11C) convex longitudinally, weakly convex transversely. Epigastric cristae (Fig. 11A, B) distinct, smooth, not cristate, separated by broad, median Y-shaped furrow; epigastric cristae just anterior of postorbital cristae, separated by short weak, furrow, very weakly sloping posterolaterally; postorbital cristae distinct, margin entire, smooth, appearing strongly concave from dorsal view, outer edge with few small granules, confluent with anterolateral margins. External orbital tooth (Fig. 11A, B) distinct, broadly triangular, outer margin straight, equal to length of inner margin, demarcated from rest of anterolateral margin by V-shaped cleft; epibranchial tooth produced, visible from dorsal view, not sharp. Anterolateral margins (Fig. 11A, B) convex, subcristate, smooth. Frontal, orbital, anterolateral, branchial, mesogastric, urogastric, cardiac, intestinal regions smooth; suborbital region (Fig. 11C) smooth with few small granules; pterygostomial regions granulose at lateral parts; subhepatic, sub-branchial regions granulose. Male pleonal somite 6 lateral margins gently sinuous in the holotype (Fig. 12B, C). G1 (Fig. 13B–E) slender, sinuous; terminal article 0.50 × length of subterminal article, strongly curving outwards, with high and broad dorsal flap on proximal half, apex medial in position, with distal margin abruptly tapered, almost perpendicularly, flap 0.62 × length of terminal article (from ventral view), slopes gradually towards tip from dorsal view; terminal, subterminal articles separated by weak dilation. G2 (Fig. 13F) long, with distal article 0.75 × length of basal article.

#### Etymology.

This species is named after its collector and discoverer, Associate Professor Sermsak Nandidarbha, formerly of Chiang Mai Rajabhat University.

#### Remarks.

The species had been referred to several times under the name; “*Thaipotamon nandidarbhi*” in local Thai social media platforms and forums. In certain instances, the name was used in conjunction with the authority “Naiyanetr, 1998” (gotoknow.org 2006). The usage of “*Thaipotamon nandidarbhi* Naiyanetr, 1998” could very likely be attributed to several local Thai newspaper outlets, which reported on the discovery of a new freshwater crab and included the name “*Thaipotamon nandidarbhi*” and with Professor Phaibul Naiyanetr being mentioned as the authority, identifying it as a new species (Daily News 1998; ThaiNews 1998; Thairath 1998). While brief descriptions of the new species were provided in the news articles, no comparisons were made to any other species (see news articles attached in and translated from Nandidarbha and Nandidarbha 2001: 123–129). Provisions of Article 13.1.1 of the ICZN (1999) Code requires that any designation of new species after 1930; “be accompanied by a description or definition that states in words characters that are purported to differentiate the taxon”. As such the name “*Thaipotamon nandidarbhi* Naiyanetr, 1998” is not available for nomenclatural purposes.

There is a scientific paper by Nandidarbha and Nandidarbha (2001), published by the National Science and Technology Development Agency (NSTDA) of Thailand, that also contains “*Thaipotamon nandidarbhi*”. The article was mainly written in Thai and available as hard copies at the time of publishing. It was only recently digitised, with open access. In the introduction to this report, it was mentioned that there exists a crab species from Soong Men (Sung Men) District of Phrae Province, locally called “Kam”, meaning dark or deep, with brief descriptions and comparisons made to the common rice field crabs in the area with regards to the size and colouration of the carapace. Within appendix A of the report, there contains a letter from Professor Phaibul Naiyanetr regarding the taxonomy of the crab from Soong Men as a new species, and that the crab will be called “*Thaipotamon nandidarbi* Naiyanetr” (Nandidarbha and Nandidarbha 2001: 119). Thereafter in appendix B, the article again states that; “This is a new species of freshwater crab found in Thailand, one that has never been reported or entered into the global taxonomy of freshwater crabs” and that “This discovery is the result of research by Associate Professor Sermsak Nandidarbha, a lecturer in the Department of Biology, Faculty of Science and Technology, Chiang Mai Rajabhat University”. According to the report, a sample of the crab was sent to a crab expert in Thailand, Professor Phaibul Naiyanetr of the Department of Biology, Faculty of Science, Chulalongkorn University, for joint examination with experts from the Netherlands. The initial examination revealed it to be a new species of freshwater crab. Professor Phaibul Naiyanetr then sent the sample to the Netherlands, with detailed documentation for publication in Crustaceana, the International Journal of Crustacean Research. The species was given the name “*Thaipotamon nandidarbhi* Naiyanetr.” (translated from Nandidarbha and Nandidarbha, 2001: 121). The name “*Thaipotamon nandidarbhi*”, however, has never been formally published as intended; the paper supposedly sent to Crustaceana reported in Nandidarbha and Nandidarbha (2001) has never been published, nor was the name published in any other journal. While the name “*Thaipotamon nandidarbhi* Naiyanetr, in Nandidarbha and Nandidarbha (2001)” was used in conjunction with some form of description and comparisons, no types were actually designated and the repository of the specimens is unknown. As a result, Article 16.4.1 of the ICZN (1999) Code, which requires a clear designation of a holotype or syntypes for the nominal taxon; and Article 16.4.2, which requires an explicit statement of intent that they will be (or are) deposited in a collection, with the name and location of that collection clearly indicated, are both not fulfilled. The name “*Thaipotamon nandidarbhi* Naiyanetr, in Nandidarbha and Nandidarbha, 2001” is thus also not available for nomenclature purposes. Although two spellings are used for the species in the report: *Thaipotamon nandidarbi* and *Thaipotamon nandidarbhi* , the spellings are likely a typographical error and here, we follow the report author’s actual name: Sermsak Nandidarbha. Therefore, the name is validated here, as *Thaipotamon nandidarbhai* sp. nov., with the necessary diagnosis and the designation of the holotype.

The distinctly hooked G1 terminal article of *Thaipotamon nandidarbhai* sp. nov., shows similar degree of curving to that in the G1 terminal articles of *T. chulabhorn*, *T. varoonphornae*, and *T. wangsaphung* sp. nov. (see later), with the distal end being perpendicular or almost perpendicular to the longitudinal axis (Figs 13B–E, 24B–E; cf. Naiyanetr 1993: fig. 1D; Ng and Naiyanetr, 1993: fig. 54B–F). This distinguishes the new species from the rest of its congeners, in which the G1 terminal article is much less strongly curved outwards, with the tip terminating at an angle not perpendicular or almost perpendicular to the longitudinal axis (~ 30–70°). *Thaipotamon nandidarbhai* sp. nov., is externally separated from *T. chulabhorn*, *T. varoonphornae*, and *T. wangsaphung* sp. nov., by the following differences: carapace appearing less strongly inflated (Fig. 11C) (vs carapace appearing more strongly inflated, Fig. 21C; cf. Naiyanetr 1993: fig. 1A; Ng and Naiyanetr 1993: fig. 20A); granulose sub-orbital, pterygostomial, sub-branchial regions (Fig. 11C) (vs regions smooth or with very few, very faint granules, Fig. 21C; cf. Naiyanetr 1993: fig. 1A; Ng and Naiyanetr 1993: fig. 20B); and male pleon somite 6 lateral margins gently sinuous (Fig. 12C) (vs male pleon somite 6 lateral margins distinctly convex in *T. varoonphornae* and *T. wangsaphung* sp. nov., Fig. 22C; cf. Ng and Naiyanetr 1993: fig. 20C). It should be noted, however, that the differences in the male pleon somite 6 lateral margins do vary slightly across among individuals. Nevertheless, *Thaipotamon nandidarbhai* sp. nov., can be further separated from *T. varoonphornae* by these additional differences: G1 terminal article relatively longer, 0.50 × length of subterminal article (Fig. 13B–E) (vs G1 terminal article relatively shorter, 0.46 × length of subterminal article; cf. Ng and Naiyanetr 1993: fig. 54B–F); dorsal flap relatively broader, 0.62 × length of terminal article (Fig. 13B–E) (vs dorsal flap relatively narrower, 0.57 × length of terminal article; cf. Ng and Naiyanetr 1993: 54B–F); and dorsal flap sloping gradually towards tip of terminal article (Fig. 13B–E) (vs dorsal flap not reaching tip of terminal article; cf. Ng and Naiyanetr 1993: 54B–F). Additionally, *Thaipotamon nandidarbhai* sp. nov., can be separated from *T. chulabhorn* and *T. wangsaphung* sp. nov., by the following differences: G1 terminal article relatively shorter, 0.50 × length of subterminal article (Fig. 13B–E) (vs relatively longer, 0.53 × length of subterminal article in *T. wangsaphung* sp. nov., and reaching 0.60 × length in *T. chulabhorn*, Fig. 23B–E; cf. Naiyanetr 1993: fig. 1D); and G1 terminal article dorsal flap distal margin more abruptly tapered, flap appearing symmetrical and semi-circular (Fig. 13B–E) (vs dorsal flap distal margin gradually tapered, appearing less symmetrical, Fig. 23B–E; cf. Naiyanetr 1993: fig. 1D).

#### Distribution.

Phrae Province, northeastern Thailand.

### Thaipotamon songkhwae sp. nov.

Taxon classificationAnimaliaDecapodaPotamidae

365E2617-0044-5FA6-9DB4-FD760170BDD7

https://zoobank.org/F8BEB528-C45F-4265-BE13-02640449BD6B

Figs 14, 15, 16

Thaipotamon phitsanulok Kumjan & Chamchoi, 2018 [nomen nudum].

#### Type material.

***Holotype***. Thailand • male (58.0 × 42.0 mm); Phitsanulok Province, Wat Bot District, Ban Yao; Chalong Nuichim leg.; Oct. 1997; ZRC 2024.0334**. *Paratype***. Thailand • 1 male (49.6 × 36.5 mm); same collection data as for holotype; ZRC 2024.0335.

#### Diagnosis.

Carapace (Fig. 14A, B) transversely sub-ovate, wider than long, CW/CL ratio 1.35–1.38, high; dorsal surface (Fig. 14C) convex longitudinally, appearing concave transversely due to inflated branchial regions. Epigastric cristae distinct, smooth, not cristate, very weakly separated by broad, median furrow, appearing almost straight; epigastric cristae just anterior of postorbital cristae, very weakly separated by furrow; postorbital cristae distinct, margin entire, smooth, appearing concave from dorsal view, outer edge smooth, confluent with anterolateral margins. External orbital tooth (Fig. 14A, B) distinct, broadly triangular, outer margin convex, equal to length of inner margin, demarcated from rest of anterolateral margin by broad cleft; epibranchial tooth very weakly produced, not clearly visible from dorsal view. Anterolateral margins (Fig. 14A, B) convex, subcristate, smooth. Frontal, orbital, anterolateral, branchial, mesogastric, urogastric, cardiac, intestinal, suborbital, pterygostomial regions (Fig. 14B, C) smooth; subhepatic, sub-branchial (Fig. 14C) regions weakly covered with striae. Male pleonal somite 6 lateral margins convex (Fig. 15C). G1 (Fig. 16B–E) broad, gently sinuous; subterminal article broad with cleft on outer margin at distal end; terminal article 0.58 × length of subterminal article, very gently curving outwards, with high, broad dorsal flap on proximal half, apex slightly skewed towards proximal portion, with relatively gradually tapered distal margin, flap 0.60 × length of terminal article (from ventral view), slopes gradually towards tip from dorsal view; terminal article separated from subterminal article by weak dilation, weakly produced collar. G2 (Fig. 16F) long, with distal article 0.63 × length of basal article.

#### Etymology.

The species is named after its local name, “Pu Song Khwae”, “Pu” meaning crab and “Song Khwae” being the local name for the type locality, Phitsanulok Province, in local Thai dialect. The name is used as a noun in apposition.

#### Remarks.

The new species, *Thaipotamon songkhwae* has been referred to frequently in various social media/forum posts, as well as in an ecotourism management paper, published in Thai (Kumjan and Chamchoi 2018), as “*Thaipotamon phitsanulok*”. The two names are most definitely referring to the same species, since all the material and accounts are from the same locality in Wat Bot District, Phitsanulok province. “*Thaipotamon phitsanulok* Kumjan & Chamchoi, 2018”, however, is not an available name. Provisions of Article 13.1.1 of the ICZN (1999) Code require that any designation of new species; “be accompanied by a description or definition that states in words characters that are purported to differentiate the taxon”, and Article 16.1 of the ICZN (1999) Code requires the names to “be explicitly indicated as intentionally new”. Since no diagnosis or comparisons were provided nor was the name explicitly stated as new, the conditions for both articles were not met. Furthermore, “*Thaipotamon phitsanulok* Kumjan & Chamchoi, 2018” also did not fulfil Articles 16.4.1 and 16.4.2 of the ICZN (1999) Code, which respectively require a clear designation of a holotype or syntypes for the nominal taxon; and an explicit statement of intent that they will be (or are) deposited in a collection, with the name and location of that collection indicated. As the conditions for all articles listed above are not fulfilled, the name “*Thaipotamon phitsanulok*” is not available for nomenclatural purposes. The *Thaipotamon* species from Wat Bot District, Phitsanulok Province is validated herein and described as *Thaipotamon songkhwae* sp. nov.

*Thaipotamon songkhwae* sp. nov., together with *T. suvankorni* sp. nov. (see below), differs from all other congeners by the G1 terminal article dorsal flap having a relatively more gradually tapered distal margin as compared to the distal margin of G1 terminal article dorsal flap of the congeners, which is more abruptly tapered and the dorsal flap apex being skewed towards the proximal portion rather than being at the median portion (Figs 13B–E, 16B–E, 19B–E, 23B–E; cf. Ng and Naiyanetr 1993: figs 51–54). Other than the different shape of G1 terminal article dorsal flap, *T. songkhwae* sp. nov., can be further differentiated from *T. siamense*, which has a similarly gently curving G1 terminal article in the following differences: G1 terminal article relatively longer, 0.58 × length of subterminal article (Fig. 13B–E) (vs G1 terminal article relatively shorter, 0.52 × length of subterminal article; cf. Ng and Naiyanetr 1993: figs 18A, 19A); and dorsal flap relatively broader, 0.60 × length of terminal article (Fig. 13B–E) (vs dorsal flap relatively narrower, 0.58 × length of terminal article; cf. Ng and Naiyanetr 1993: figs 18A, 19A).

*Thaipotamon songkhwae* sp. nov., most closely resembles *T. suvankorni* sp. nov., in terms of G1 morphology. However, *Thaipotamon songkhwae* sp. nov., can be distinguished from *T. suvankorni* sp. nov., by the following differences: carapace relatively more transverse, ~ 1.35–1.38 × broader than long (Fig. 14A, B) (vs carapace relatively less transverse, ~ 1.20–1.30 × broader than long, Fig. 17A, B); external orbital tooth relatively more broadly triangular (Fig. 14B) (vs external orbital tooth relatively more acutely triangular, Figs 17B, 20A); cervical grooves and grooves between epibranchial and postorbital cristae indistinct (Fig. 14A, B) (vs cervical grooves and grooves between epibranchial and postorbital cristae shallow but distinct, Figs 17B, 20A); G1 subterminal article with cleft on outer margin at distal end (Fig. 16B) (vs G1 subterminal article without cleft on outer margin, Fig. 19B); G1 terminal article relatively longer, 0.58 × length of subterminal article (Fig. 16B–E) (vs G1 terminal article relatively shorter, 0.54 × length of subterminal article, Fig. 19B–E); G1 terminal article relatively less curved outwards (Fig. 16B–E) (vs G1 terminal article relatively more strongly curved outwards; Fig. 19B–E); G1 terminal article tapers to narrow, pointed end (Fig. 16B–E ) (vs G1 terminal article tapers to broader, rounded end, Fig. 19B–E); G1 terminal article dorsal flap relatively broader, 0.60 × length of terminal article (Fig. 16D) (vs G1 terminal article dorsal flap relatively narrower, 0.57 × length of terminal article, Fig. 19D); and G1 terminal article dorsal flap extends all the way to tip of terminal article (Fig. 16D) (vs G1 terminal article dorsal flap not reaching tip of terminal article, Fig. 19D).

#### Distribution.

Phitsanulok Province, northern Thailand.

### Thaipotamon suvankorni sp. nov.

Taxon classificationAnimaliaDecapodaPotamidae

CD301756-6AC4-5818-9858-3D1BA0FB9A5D

https://zoobank.org/19EC85AA-3C2F-437E-B5DA-D97B181B71F8

Figs 17, 18, 19, 20

#### Type material.

***Holotype***. Thailand • male (45.5 × 35.0 mm); Chachoengsao Province, Tha Ta Kiep Wildlife Sanctuary; Ubonwan Booncharm leg.; 26 May 1993; ZRC 2024.0336***. Paratypes***. Thailand • 1 male (38.7 × 29.9 mm); 2 females (41.3 × 32.3, 36.4 × 30.4 mm); same collection data as for holotype; ZRC 2024.0337.

#### Diagnosis.

Carapace (Figs 17A, 17B, 20A) transversely sub-ovate, wider than long, CW/CL ratio 1.20–1.30, high; dorsal surface (Fig. 17C) convex transversely and longitudinally. Epigastric cristae (Fig. 17C) distinct, smooth, not cristate, separated by broad, median Y-shaped furrow, gently sloping posterolaterally; epigastric cristae just anterior of postorbital cristae, separated by short weak, furrow; postorbital cristae distinct, margin entire, smooth, appearing weakly concave from dorsal view, outer edge smooth, confluent with anterolateral margins. External orbital tooth (Fig. 17B) distinct, narrowly triangular, outer margin straight, equal to length of inner margin, demarcated from rest of anterolateral margin by V-shaped cleft; epibranchial tooth (Fig. 17B) small, visible from dorsal view as nodule. Anterolateral margins convex, not cristate, smooth. Frontal, orbital, anterolateral, branchial, mesogastric, urogastric, cardiac, intestinal, suborbital, pterygostomial regions smooth; subhepatic, sub-branchial regions (Fig. 17C) covered with weak striae. Male pleonal somite 6 lateral margins convex (Fig. 18C). G1 (Fig. 19B–E) slender, sinuous; terminal article 0.54 × length of subterminal article, gently curving outwards, with high and broad dorsal flap on proximal half, apex slightly skewed towards proximal portion, with relatively gradually tapered distal margin, flap 0.57 × length of terminal article (from ventral view), not reaching tip from dorsal view; terminal article not distinctly separated from subterminal article by dilation. G2 (Fig. 19F) long, with distal article 0.65 × length of basal article. Female vulvae (Fig. 20C) large, oval, recessed, opening directed mesially, located at mesial end of sternite 6, approximtely half the length of sternite 6, with short, calcified eave and soft membranous flap-like protrusion.

#### Etymology.

This species is named in honour of Mr. Phairot Suvankorn, former director of the Department of Royal Forestry, Ministry of Agriculture, Thailand.

#### Remarks.

The general G1 structure of *Thaipotamon suvankorni* sp. nov., resembles that of *T. songkhwae* sp. nov. They can nevertheless be separated by other G1 characters, as well as some carapace characters (see earlier remarks for *Thaipotamon songkhwae* sp. nov.). *Thaipotamon suvankorni* sp. nov., is also similar to *T. siamense* in the relatively weakly curved G1 terminal article; however, *T. suvankorni* sp. nov., still clearly differs from *T. siamense* by the following differences: G1 terminal article relatively longer, 0.54 × length of subterminal article (Fig. 19B–E) (vs G1 terminal article relatively shorter, 0.52 × length of subterminal article; cf. Ng and Naiyanetr 1993: figs 18A, 19A); and G1 terminal article dorsal flap apex appears slightly skewed towards proximal portion, with relatively gradually tapered distal margin (Fig. 19D) (vs G1 terminal article dorsal flap apex medial, with abruptly tapered distal margin; cf. Ng and Naiyanetr 1993: fig. 19A). Since female specimens are available for this species, vulvae (Fig. 20C) characters, which are potentially diagnostic, have also been included in the Diagnosis for future taxonomic reference.

#### Distribution.

Chachoengsao Province, eastern Thailand.

### Thaipotamon wangsaphung sp. nov.

Taxon classificationAnimaliaDecapodaPotamidae

7413DBF9-38DF-5888-885F-E85507407428

https://zoobank.org/0B03B44A-284D-4D96-BC69-6724CB2A81E5

Figs 21, 22, 23, 24

#### Type material.

***Holotype***. Thailand • male (40.7 × 28.7 mm); Loei Province, Wang Saphung District, Ban Na Luang, Phu Luang; P. Naiyanetr leg.; 14 Oct. 1998; ZRC 2024.0338**. *Paratypes***. Thailand • 1 male (36.6 × 27.0 mm); 1 female (33.0 × 24.9 mm); same collection data as for holotype; ZRC 2024.0339.

#### Diagnosis.

Carapace (Figs 21A, 21B, 24A) transversely sub-ovate, wider than long, CW/CL ratio 1.33–1.37, high; dorsal surface (Fig. 21C) convex longitudinally and transversely. Epigastric cristae distinct, smooth, not cristate, separated by broad, median Y-shaped furrow, sloping posterolaterally strongly; epigastric cristae anterior of postorbital cristae, barely separated by furrow; postorbital cristae distinct, margin entire, smooth, appearing concave from dorsal view, outer edge smooth, confluent with anterolateral margins. External orbital tooth (Fig. 21B) distinct, narrowly triangular, outer margin straight, subequal to length of inner margin, demarcated from rest of anterolateral margin by V-shaped cleft; epibranchial tooth (Fig. 21B) weakly produced, visible from dorsal view as nodule. Anterolateral margins convex, smooth. Frontal, orbital, anterolateral, branchial, mesogastric, urogastric, cardiac, intestinal, suborbital, pterygostomial regions smooth; subhepatic, sub-branchial regions (Fig. 21C) weakly granulose. Male pleonal somite 6 lateral margins convex (Fig. 22C). G1 (Fig. 23B–E) slender, sinuous; terminal article 0.53 × length of subterminal article, strongly curving outwards, with high and broad dorsal flap on proximal half, apex medial in position, with proximal margin abruptly tapered, almost perpendicularly, with relatively gradually tapered distal margin, flap 0.57 × length of terminal article (from ventral view), reaching tip from dorsal view; terminal article not distinctly separated from subterminal article by dilation. G2 (Fig. 23F) long, with distal article 0.61 × length of basal article. Female vulvae (Fig. 24C) large, semi-circular, recessed, opening directed mesially, located at mesial end of sternite 6, more than half length of sternite 6, with calcified eave and large, soft membranous flap-like protrusion, obscuring opening.

#### Etymology.

The species name is named after the locality it was collected from, Wang Saphung District in Loei Province, northeastern Thailand. The name is used as a noun in apposition.

#### Remarks.

*Thaipotamon wangsaphung* sp. nov., most closely resembles *T. holthuisi* Naiyanetr & Yeo, 2010, in its G1 terminal article, which features a high, broad dorsal flap on proximal half, with apex in median position, distal portion bent outwards (Fig. 23B–E; cf. Naiyanetr and Yeo 2010: fig. 2). *Thaipotamon wangsaphung* sp. nov., can nevertheless be distinguished from *T. holthuisi* by the following characters: epigastric cristae separated from postorbital cristae by shallow grooves (Figs 21B, 24A) (vs epigastric cristae appearing to be confluent with postorbital cristae; cf. Naiyanetr and Yeo 2010: fig. 1A); epigastric cristae sloping posterolaterally in dorsal view (Figs 21B, 24A) (vs epigastric cristae parallel to frontal margin in dorsal view; cf. Naiyanetr and Yeo 2010: fig. 1A); postorbital cristae concave in dorsal view (Figs 21B, 24A) (vs postorbital cristae subparallel to frontal margin in dorsal view; cf. Naiyanetr and Yeo 2010: fig. 1A); G1 terminal article relatively longer, 0.53 × length of subterminal article (Fig. 23B–E) (vs G1 terminal article relatively shorter, 0.51 × length of subterminal article; cf. Naiyanetr and Yeo 2010: fig. 2); and G1 terminal article dorsal flap distal margin gently tapering with a long, broad, gently convex apex (Fig. 23D) (vs G1 terminal article dorsal flap distal margin abruptly tapered, almost perpendicularly; cf. Naiyanetr and Yeo 2010: fig. 2C). Since a female specimen is available for this species, vulvae (Fig. 24C) characters, which are potentially diagnostic, have also been included in the Diagnosis for future taxonomic reference.

#### Distribution.

Loei Province, northeastern Thailand.

##### Key to adult male *Thaiphusa* species

**Table:** 

1	G1 terminal article not distinctly bent outwards, almost straight	**2**
–	G1 terminal article distinctly bent and curving outwards	**4**
2	Carapace with branchial regions not distinctly inflated; G1 subterminal article proximal part abruptly tapering to distal neck-like part with distinct L-shaped cleft on lateral margin	***T. tenasserimensis* (De Man, 1898)** [Thagata, Mount Mooleyit, southern Myanmar]
–	Carapace with branchial regions distinctly inflated, appearing swollen; G1 subterminal article proximal part tapering relatively gentler to distal neck-like distal part, without trace of cleft on lateral margins	**3**
3	Male thoracic sternite 3/4 groove demarcating suture not distinct; G1 terminal article longer, 0.63 × length of subterminal article, with dorsal flap lower and more gently convex; G1 subterminal article with narrowed, neck-like distal part longer than expanded proximal	***T. sirikit* (Naiyanetr, 1992)** [Kanchanaburi, western Thailand]
–	Male thoracic sternite 3/4 groove demarcating suture prominent; G1 terminal article shorter, 0.52 × length of subterminal article; G1 subterminal article with slender distal part relatively shorter, subequal to or shorter than expanded proximal part	***T. reginamimus* sp. nov**. [Kanchanaburi, western Thailand]
4	G1 terminal article distal end more strongly curving outwards, nearly parallel to horizontal axis, dorsal flap with broad, gently convex apex; subterminal article without broad angular cleft on outer margin	***T. mongkol* sp. nov**. [Kanchanaburi, western Thailand]
–	G1 terminal article distal end less strongly curving outwards, ~ 40° from horizontal axis, dorsal flap with bluntly angular apex; subterminal article with broad angular cleft on outer margin	***T. chantaburiensis* (Chuensri, 1973)** [Chanthaburi, eastern Thailand]

##### Key to adult male *Thaipotamon* species

**Table:** 

1	Dorsal flap of G1 terminal article with apex skewed towards proximal portion	**2**
–	Dorsal flap of G1 terminal article with apex in median portion	**3**
2	Carapace more transverse, ~ 1.35–1.38 × broader than long; external orbital tooth relatively more broadly triangular; cervical grooves and grooves between epibranchial and postorbital cristae indistinct; G1 subterminal article with cleft on outer margin at distal end; G1 terminal article relatively longer, 0.58 × length of subterminal article, tip narrow and sharp, with broad dorsal flap, 0.60 × length of terminal article, completely extending to tip of terminal article	***T. songkhwae* sp. nov**. [Phitsanulok, northern Thailand]
–	Carapace less transverse, ~ 1.31 × broader than long; external orbital tooth relatively more acutely triangular; cervical grooves and grooves between epibranchial and postorbital cristae shallow but distinct; G1 subterminal article without cleft on outer margin; G1 terminal article relatively shorter, 0.54 × length of subterminal article, tip broad and blunt, with narrower dorsal flap, 0.57 × length of terminal article, not reaching tip of terminal article	***T. suvankorni* sp. nov**. [Chachoengsao, eastern Thailand]
3	Dorsal flap of G1 terminal article not reaching tip of terminal article	**4**
–	Dorsal flap of G1 terminal article reaching tip of terminal article	**7**
4	Cervical grooves and grooves between epibranchial and postorbital cristae shallow but distinct; epistome posterior margin median tooth acutely triangular; G1 relatively more strongly curved outwards, tip terminating parallel or almost parallel to horizontal axis	**5**
–	Cervical grooves and grooves between epibranchial and postorbital cristae indistinct; epistome posterior margin median tooth broadly triangular; G1 relatively less strongly curved outwards, tip directed between 45–70° from horizontal axis	**6**
5	External orbital tooth very acutely triangular; epistome posterior margin lateral parts straight; G1 terminal article longer, 0.59 × length of subterminal article, dorsal flap broader, 0.62 × length of terminal article	***T. chulabhorn* Naiyanetr, 1993** [Maha Sarakham, northeastern Thailand]
–	External orbital tooth more broadly triangular; epistome posterior margin lateral parts gently convex; G1 terminal article shorter, 0.46 × length of subterminal article, dorsal flap narrower, 0.57 × length of terminal article	***T. varoonphornae* Ng & Naiyanetr, 1993** [Sa Kaeo, eastern Thailand]
6	Carapace more transverse, ~ 1.40 × broader than long; epigastric cristae lower, less distinct; supra- and infraorbital margins more strongly cristate; epibranchial tooth relatively sharper; G1 terminal article relatively longer, 0.51 × length of terminal article, with tip directed laterally, dorsal flap narrower, 0.62 × of the terminal article; distal part of outer margin of G1 subterminal article with shallow, broad rectangular cleft	***T. dansai* Ng & Naiyanetr, 1993** [Loei, northeastern Thailand]
–	Carapace relatively less broad, ~ 1.35 × broader than long; epigastric cristae stronger, more distinct; supra- and infraorbital margins less distinctly cristate; epibranchial tooth blunt, knob-like; G1 terminal article relatively shorter, 0.49 × length of terminal article, with tip not directed laterally but gently upcurved instead, dorsal flap broader, 0.65 × of terminal article; distal part of outer margin of G1 subterminal article without cleft	***T. lomkao* Ng & Naiyanetr, 1993** [Phetchabun, north-central Thailand]
7	Sub-branchial and pterygostomial regions granulose to slightly granulose; groove demarcating suture between anterior thoracic sternites 3 and 4 well defined, complete	**8**
–	Sub-branchial regions smooth or very slightly, granulose, pterygostomial regions smooth; groove demarcating suture between anterior thoracic sternites 3 and 4 weak [unknown in *T. smitinandi*]	**10**
8	Carapace dorsal surface appears less strongly inflated, sub-branchial regions granulose; lateral margins of male pleonal somite 6 gently sinuous, gently convex posteriorly to slightly concave distally	***T. nandidarbhai* sp. nov**. [Phrae, northeastern Thailand]
–	Carapace dorsal surface appears more strongly inflated, sub-branchial regions with few, very faint granules; lateral margins of male pleonal somite 6 distinctly convex	**9**
9	Epigastric cristae separated from postorbital cristae by shallow grooves; postorbital cristae strongly concave in dorsal view; G1 terminal article relatively longer, 0.53 × length of subterminal article; G1 dorsal flap distal margin gently tapering with long, broad, gently convex apex	***T. wangsaphung* sp. nov**. [Loei Province, northeastern Thailand]
–	Epigastric cristae appearing to be confluent with postorbital cristae; postorbital cristae subparallel to frontal margin in dorsal view; G1 terminal article relatively shorter, 0.51 × length of subterminal article; G1 dorsal flap distal margin abruptly tapered, almost perpendicularly	***T. holthuisi* Naiyanetr & Yeo, 2010** [Phetchabun, north-central Thailand]
10	Carapace relatively less transverse, 1.33 × broader than long; G1 terminal article relatively longer, 0.52 × length of subterminal article, slenderer, ~ 4.8 × longer than proximal width, dorsal flap less broad, 0.58 × length of terminal article; G1 subterminal article relatively narrower, without cleft on outer margin	***T. siamense* (A. Milne-Edwards, 1869)** [“Bangkok, Siam”, central Thailand]
–	Carapace relatively more transverse, 1.37 × broader than long; G1 terminal article relatively shorter, 0.50 × length of subterminal article, stouter, ~ 4.0 × longer than proximal width, dorsal flap broader, 0.61 × length of terminal article; G1 subterminal article relatively broader, with weak but distinct cleft on upper part of outer margin	***T. smitinandi* (Naiyanetr & Türkay, 1984)** [Chanthaburi, eastern Thailand]

## Supplementary Material

XML Treatment for Thaiphusa reginamimus

XML Treatment for Thaiphusa mongkol

XML Treatment for Thaipotamon nandidarbhai

XML Treatment for Thaipotamon songkhwae

XML Treatment for Thaipotamon suvankorni

XML Treatment for Thaipotamon wangsaphung

## Additional information

### Artificial Intelligence (AI) use

The authors accept full responsibility for the content of the manuscript, including the disclosure of any use of AI.

No AI tools were used in the preparation of this manuscript.

### Author ORCIDs

Z.W. Tan https://orcid.org/0000-0002-9878-6958

D.C.J. Yeo https://orcid.org/0000-0003-2669-4349

### Data availability

All of the data that support the findings of this study are available in the main text.
